# Anthropogenic Water Withdrawals Modify Freshwater Inorganic Carbon Fluxes across the United States

**DOI:** 10.1021/acs.est.4c09426

**Published:** 2025-02-17

**Authors:** Elizabeth M. Flint, Matthew J. Ascott, Daren C. Gooddy, Mason O. Stahl, Ben W. J. Surridge

**Affiliations:** † British Geological Survey, Maclean Building, Crowmarsh, Wallingford, Oxfordshire OX10 8BB, U.K.; ‡ Lancaster Environment Centre, Lancaster University, Lancaster LA1 4YQ, U.K.; § UK Centre for Ecology and Hydrology, Maclean Building, Crowmarsh, Wallingford, Oxfordshire OX10 8BB, U.K.; ∥ Department of Geosciences, Union College, Schenectady, New York 12308, United States

**Keywords:** dissolved inorganic carbon, carbon dioxide emissions, freshwater withdrawals, biogeochemical cycling, carbon budgets

## Abstract

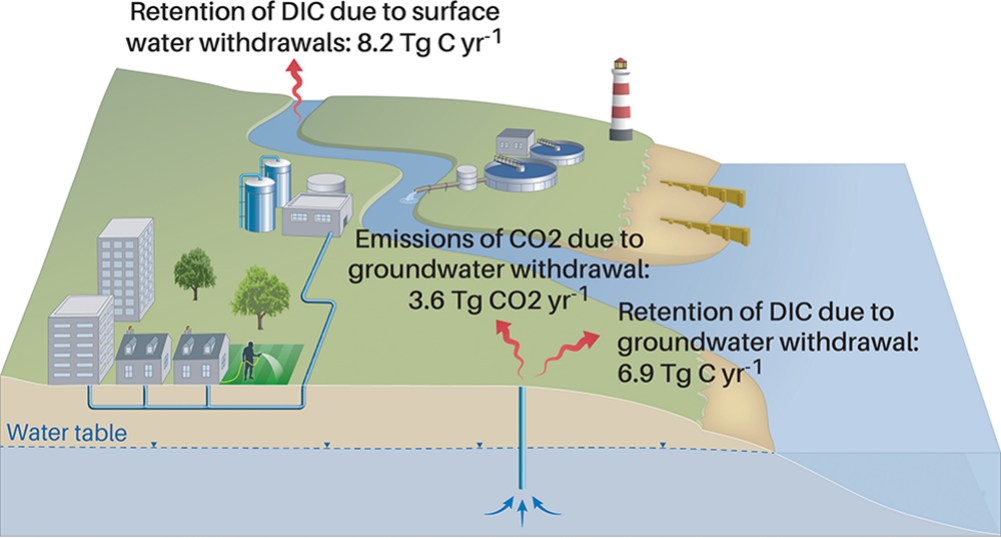

Quantifying inorganic carbon fluxes to and from freshwater environments is essential for the accurate determination of the total amount of carbon exported to both the atmosphere and oceans. However, understanding of how anthropogenic freshwater withdrawals perturb land-freshwater-ocean and freshwater-atmosphere inorganic carbon fluxes is limited. Using the United States (US) as an exemplar, we estimate that fresh surface water withdrawals across the country during the year 2015 resulted in a median gross dissolved inorganic carbon (DIC) retention flux of 8.2 (uncertainty range: 6.7–9.9) Tg C yr^–1^, equivalent to 28.3% of the total export of DIC to the oceans from US rivers. The median gross retention flux due to fresh groundwater withdrawals was 6.9 (uncertainty range: 5.3–8.8) Tg C yr^–1^, over eight times the magnitude of the DIC flux to the oceans by US subterranean groundwater discharge. The degassing of CO_2_ supersaturated groundwater following withdrawal emitted 3.6 (uncertainty range: 2.2–5.5) Tg of CO_2_ yr^–1^, 112% larger than previous estimates. On a county level, these CO_2_ emissions exceeded CO_2_ emissions from major emitting facilities across 45% of US counties. Reported results and a data analysis framework have important implications for the accurate development of carbon budgets across the US and around the world.

## Abstract 2

Understanding of how freshwater withdrawals perturb inorganic carbon cycling is limited. We estimate the impact of freshwater withdrawals on freshwater dissolved inorganic carbon retention and the CO_2_ emissions from degassing groundwater withdrawals across the United States, with results having implications for accurately determining carbon budgets worldwide.

## 1 Introduction

Fresh waters are critical, reactive interfaces that influence the transport and fate of carbon (C).^1^ Accurately estimating fluxes of the multiple chemical and physical species of C (dissolved, particulate, inorganic, and organic) to and from freshwater environments is essential for understanding the quality of potable water, ecosystem functioning, and the role of fresh waters in the transfer of different C fractions between terrestrial, atmospheric and oceanic systems.^1−3^ The delivery of both organic and inorganic C to the oceans by rivers and subterranean groundwater flow, as well as the burial of organic C within freshwater sediments and outgassing of CO_2_ from fresh waters to the atmosphere, have been estimated on global^1,4−7^ and continental scales.^3,8,9^ However, despite growing recognition of fresh waters as critical interfaces that moderate the global C cycle,^10^ many processes with the potential to perturb C fluxes remain poorly constrained, particularly those associated with groundwater and anthropogenic activities. Human activities, including climate and landscape change and the construction of reservoirs, can impact C burial, outgassing, and export.^11−13^ While some research has focused on developing a more integrated understanding of freshwater C cycling,^14,15^ the continued omission of these anthropogenic influences within C budgets can lead to biased estimation and associated uncertainty of other C balance components.^7,11,16−18^ This may hinder the development of the robust and integrated C budgets that are necessary to inform policies that are able to respond effectively to a changing C cycle.^18^

Freshwater withdrawals are defined by the United States Geological Survey (USGS) as “the total amount of water removed from the water source for a particular use”,^19^ with these sources being most commonly either a groundwater well or surface water intake. Recent research has identified the anthropogenic withdrawal of fresh water as a potentially significant mechanism perturbing C cycling in fresh waters. Globally, withdrawals of groundwater were estimated to bring 19 Tg C yr^–1^ to surface water environments,^20^ with 70% of this flux (13.3 Tg C yr^–1^) being in the form of dissolved inorganic carbon (DIC). The degassing of CO_2_ supersaturated groundwaters upon their equilibration with the atmosphere^21−25^ and the treatment of organic C within withdrawn fresh water prior to distribution^26^ are identified as sources of atmospheric CO_2_ around the world. Reservoir drawdown areas, which are hotpots for emissions of CO_2_ to the atmosphere, can also be created in part due to anthropogenic water withdrawal.^27^ Freshwater withdrawals have also been found to prevent the downstream export of organic C to the oceans by rivers.^26,28^ Despite these findings, an integrated understanding of the impact that both fresh groundwater and surface water withdrawals can have on C cycling, either nationally or globally, is yet to be developed. Perturbations to the C cycle continue to generate increased risks of tipping over a range of planetary boundaries.^29^ Addressing this gap in understanding is therefore increasingly urgent and the focus of the research reported here.

Fluxes of total dissolved C from United States (US) fresh waters to the ocean are predominantly in the form of DIC.^30^ The country has some of the highest DIC exports to the ocean of anywhere globally,^6,17^ with the Mississippi River making the largest individual contribution (17.39 Tg C yr^–1^)^30^ to the total amount of DIC exported by US rivers to the oceans (29 Tg C yr^–1^).^3^ The US also has some of the highest total and per capita withdrawals of fresh water in the world.^31^ The removal of this water from both groundwater and surface water environments has been identified as an important inorganic nitrogen retention mechanism^32^ and a nationally significant source of CO_2_ emissions.^21,25^ In the research reported here, the US is used as an exemplar to develop and apply a new framework in order to quantify the impacts of both groundwater and surface withdrawals on freshwater DIC fluxes. We hypothesize that

1. Surface water and groundwater withdrawals will perturb lateral dissolved inorganic carbon fluxes within freshwater environments across the United States
2. Degassing fresh groundwater withdrawals will act as locally important sources of atmospheric CO_2_ that will vary spatially and by water use sector across the United States

These hypotheses are addressed using a range of publicly available data sets including freshwater withdrawal volumes and DIC concentrations. The implications of these US-based findings for global C cycling and future research needs in this area are discussed.

## 2 Materials and Methods

### 2.1 Estimating the Gross Impact of Withdrawals of Fresh Water on the Lateral Export of DIC

The gross fluxes of DIC removed from fresh waters due to groundwater and surface water withdrawals (WD-DIC_gw_ and WD-DIC_sw_, in Tg C yr^–1^) were estimated for each county across the US as the product of county-level fresh groundwater and surface water withdrawal volumes for each major water use sector (WD_gw_ and WD_sw_, in L yr^–1^) during the year 2015,^33^ and median county-level groundwater and surface water DIC concentrations (DIC_gw_ and DIC_sw_, in Tg C L^–1^,eqs 1 and 2).

The workflow developed in this research for obtaining DIC_gw_ and DIC_sw_ concentrations is outlined in Supplementary Note 1. Concentrations of DIC are infrequently measured during water quality monitoring, and so, all water quality parameter queries were extended to be between 01.01.2010 and 31.12.2020. Data retrievals from the Water Quality Portal^34^ returned no measured DIC data for groundwater sites and measured surface water DIC data for only 79 counties. Given this lack of measured DIC concentration data, THINCARB (Thermodynamic modeling of Inorganic CARBon) was used to model DIC_gw_ and DIC_sw_ concentrations.^35^ Model inputs were queried using the advanced search tool within the Water Quality Portal.^34^ More specifically, alkalinity (from filtered samples), pH, water temperature, altitude, and calcium concentrations were queried as characteristics (Table S1). This facilitated the return of values from both Environmental Protection Agency (EPA) and USGS databases, as well as state, federal, tribal, and local agencies. This enabled the modeling of DIC_gw_ and DIC_sw_ concentrations across 1,024 and 584 counties, respectively (Figures S1 and S2).
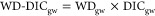
1
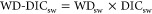
2Where input data required for modeling DIC concentrations was not available, equilibrium equations,^36^ using measured pH and the measured concentration of either carbonate or bicarbonate (CO_3_^2–^ or HCO_3_^–^) from groundwaters and surface waters were used to calculate DIC_gw_ and DIC_sw_ concentrations for a further 463 and 188 counties, respectively (Figures S1 and S2). Measured values of pH and CO_3_^2–^ and HCO_3_^–^ concentrations were queried as characteristics using the Water Quality Portal’s advanced search tool.^34^ For the 1621 and 2223 counties without sufficient input data to model or calculate DIC_gw_ and DIC_sw_ values, respectively, median state-level DIC concentrations (derived from THINCARB modeling) were applied to the state’s constituent county (Figures S1 and S2). It should be noted that DIC concentration data was not able to be linked to specific withdrawals from individual groundwater wells or surface water intakes, due to the fact that withdrawal data is provided on a county-level resolution.^33^ The limitations associated with acquiring concentration data using these various approaches are discussed in Supplementary Note 1. County-level fluxes were aggregated to give a national-level total and lower and upper estimates for all fluxes were made by applying a ± 10% uncertainty on withdrawal volumes^37^ and using 25th and 75th percentile DIC concentrations within eqs 1 and 2, respectively.^38^ County-level fluxes were also normalized for land area in kg C km^–2^ yr^–1^.

### 2.2 Estimating the Net Impact of Freshwater Withdrawals on the Lateral Export of DIC

Fully understanding the impact of freshwater withdrawals on C cycling requires the fate of the withdrawn DIC to be determined. The research reported here attempts to estimate net withdrawal DIC fluxes by assessing the key processes that may affect the speciation and flux of DIC returned to fresh waters following withdrawal. The sources of data and assumptions used to make initial estimates of net WD-DIC fluxes for major US water use sectors are shown in Table S2, with the methodology detailed in Supplementary Note 2. These net fluxes can be defined as the flux of DIC that is permanently prevented from downstream transport on time scales relevant to overall C budgets, due to either groundwater or surface water withdrawal, having accounted for speciation changes, return flows, and consumption. A positive net WD-DIC flux indicates DIC retention from the fresh surface water or groundwater system, whereas a negative flux denotes a net contribution of DIC to fresh water. As a hypothetical example, if DIC was removed exclusively from groundwater via withdrawals but was then returned entirely to surface water after use via effluent discharge, this would result in a positive net WD-DIC_gw_ flux and a negative net WD-DIC_sw_ flux.

Data were not available for a range of additional key processes, including water consumption and key chemical processes involving DIC within the industry and the volume of irrigation and mining return flows to both surface water and groundwater. This meant that net WD-DIC_sw_ flux estimates for irrigation, public supply, industrial and mining water use sectors, and net WD-DIC_gw_ flux estimates for irrigation and mining water use sectors, could not be made at this time (Figure 2).

### 2.3 Estimating CO_2_ Emissions Associated with Degassing Groundwater Withdrawals

County-level emissions of CO_2_ due to the degassing of CO_2_ supersaturated groundwater withdrawals (WD-CO_2 gw_, in kg CO_2_ yr^–1^) were estimated as the product of county-level fresh groundwater withdrawal volumes (WD_gw_, in L yr^–1^) and median county-level excess CO_2_ concentrations of groundwater when in equilibrium with the atmosphere (E[CO_2 gw-atm_]; eq 3). E[CO_2 gw-atm_] concentrations were estimated using excess CO_2_ partial pressures (EpCO_2_) modeled by THINCARB using inputs described in Section 2.1. EpCO_2_ is the ratio of the CO_2_ partial pressure in the groundwater sample (pCO_2 gw_) to the CO_2_ partial pressure of the atmosphere (pCO_2 atm_), which was assumed to be 0.0003994 atm for the year 2015 (eq 4).
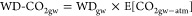
3
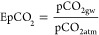
4

This methodology assumes the rate of CO_2_ degassing from supersaturated groundwaters to be faster than the rate of groundwater’s return to aquifers after use, as well as the full equilibration of groundwater with the atmosphere.^39,40^ The impact of these assumptions upon WD-CO_2 gw_ fluxes is further investigated within Supplementary Note 4. EpCO_2_ values were subsequently used to determine E[CO_2 gw-atm_] concentrations using eqs 5–8. The use of the Van’t Hoff equation allowed changes in temperature (*T*) to be related to changes in equilibrium constant (*K*_H_; eq 5). pCO_2 atm_ values were then corrected using *K*_H_ to give the partial pressure of groundwater when it was in equilibrium with the atmosphere (pCO_2 gw-atm_; eq 6). pCO_2 gw_ was then estimated as the product of EpCO_2_ and pCO_2 gw-atm_ (eq 7). Finally, excess concentrations of CO_2_ in groundwater samples (E[CO_2 gw-atm_]), in mg CO_2_ L^–1^, were determined as the difference between pCO_2 gw_ and pCO_2 gw-atm_ (eq 8). Median state-level E[CO_2 gw-atm_] concentrations were applied to the 2,084 counties without a modeled EpCO_2_ value. See Supplementary Data 1 for full calculations.
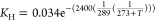
5
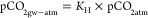
6
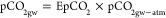
7
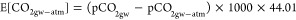
8

### 2.4 Contextualizing the Magnitude of Gross Freshwater Withdrawal DIC Fluxes and Groundwater Withdrawal CO_2_ Emissions

The magnitude of the gross national-level WD-DIC_sw_ flux was contextualized through its comparison with the national-level DIC flux from US fresh surface waters, which is the sum of lateral DIC export to the oceans and the outgassing of CO_2_ from rivers and lakes.^3^ The gross national-level WD-DIC_gw_ flux was compared to the subterranean groundwater discharge DIC flux to the oceans (DIC_SGD_) across the US (Table 1). The DIC_SGD_ flux (0.7 Tg C yr^–1^) was estimated as the product of the annual US fresh subterranean groundwater discharge volume (1.5 × 10^13^ L yr^–1^),^41^ and the median DIC_gw_ concentration determined in this research (48.2 mg C L^–1^). The contribution that withdrawals from each individual water use sector make to these gross withdrawal fluxes was also assessed.

**Table 1:** Gross Freshwater Withdrawal Dissolved Inorganic Carbon Fluxes for Groundwater and Surface Water (WD-DIC_gw_ and WD-DIC_sw_), Expressed As a Percentage, Compared to Other Components of the Freshwater Carbon Cycle across the United States

flux (Tg C yr^–1^)	outgassing of CO_2_ by rivers and lakes (85.3)^a^	river DIC export to the ocean (29)^a^	total surface water export DIC flux (114.3)^a^	subterranean groundwater discharge DIC flux to the ocean (0.7)^b^	total DIC flux to the ocean by rivers and groundwater (29.7)
WD-DIC_sw_ (6.7–9.9)	7.9–11.6	23.1–34.1	5.9–8.7		
WD-DIC_gw_ (5.3–8.8)				757–1257	
WD-DIC_total_ (12.0–18.7)					40.4–63.0

a Butman, Stackpoole, Stets, McDonald, Clow and Striegl.^3^

b Estimated using the total subterranean groundwater discharge estimate made by Sawyer, David, and Famiglietti^41^ and the median DIC concentration of groundwater determined in this study (48.2 mg C L^–1^).

The potential importance of the national-level WD-CO_2 gw_ flux was evaluated through its comparison with the estimated CO_2_ emissions from US rivers and lakes.^3^ WD-CO_2 gw_ fluxes due to irrigation and public supply water use sectors were also compared to other sector-specific CO_2_ emissions, including those from agricultural liming practices^42^ and the electricity generation for both the pumping of groundwater for irrigation^43^ and the operation of drinking water systems.^44^ Counties with significant WD-CO_2 gw_ emissions were identified through the comparison of county-level WD-CO_2 gw_ estimates to the county’s total CO_2_ emissions from major sources,^45^ specifically, those sources obliged to report to the US EPA’s Greenhouse Gas Reporting Program (GHGRP-CO_2_). These detailed emissions data are collected from approximately 7300 greenhouse gas emitting facilities across the US that emit over 25,000 t of CO_2_ yr^–1^, either via combustion or process emissions. When combined, these emissions account for around 50% of total US greenhouse gas emissions.^46^

## 3 Results

### 3.1 Impact of Withdrawals of Fresh Water on Lateral DIC Fluxes

#### 3.1.1 Gross DIC Carbon Fluxes Associated with Withdrawals of Fresh Water

**Figure 1:**
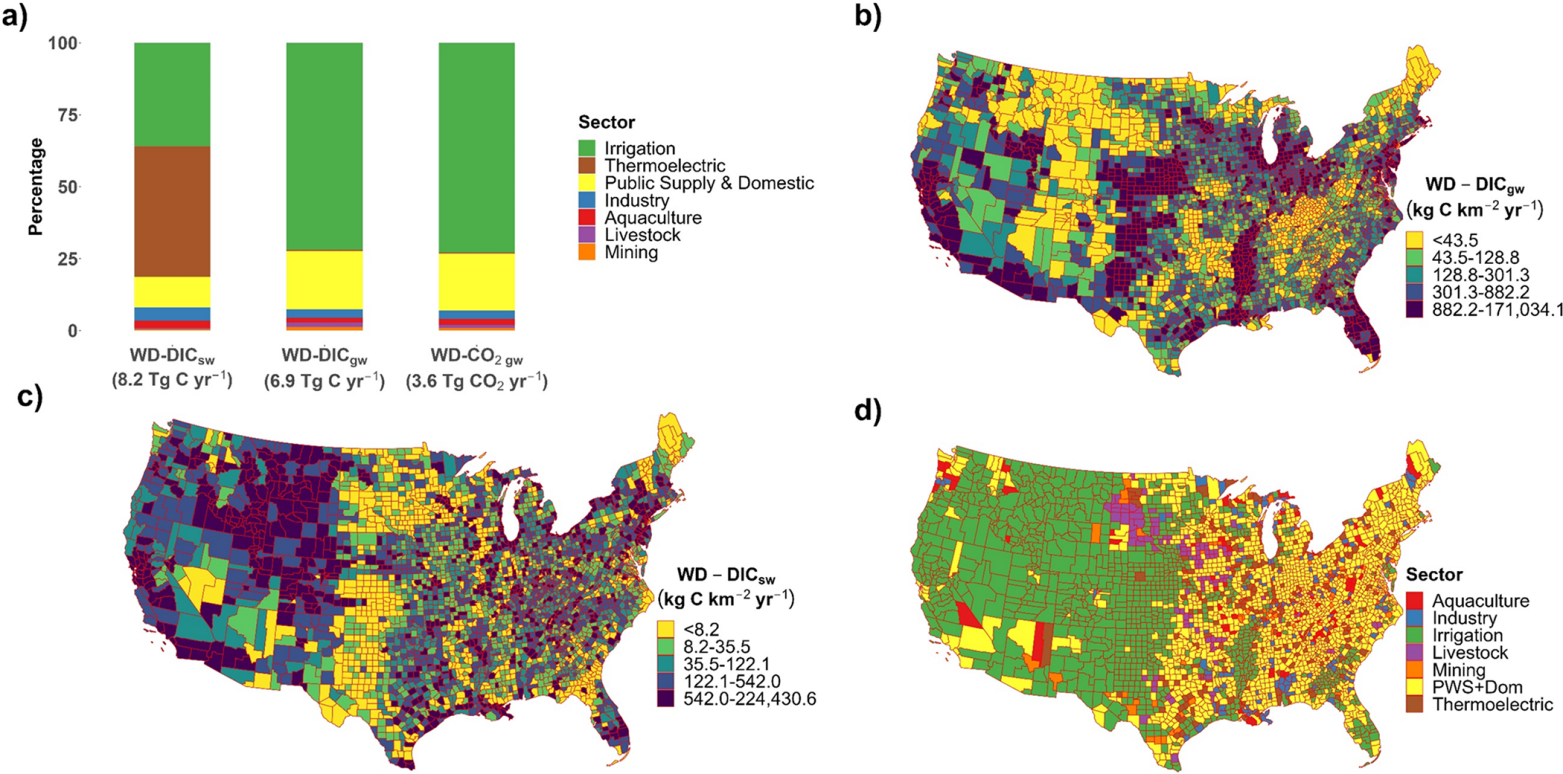
Freshwater withdrawal dissolved inorganic carbon flux (WD-DIC) estimates across the United States. (a) Contribution of water use sector withdrawals to gross national-level surface water and groundwater withdrawal DIC fluxes (WD-DIC_sw_ and WD-DIC_gw_), and the national-level emissions of CO_2_ due to degassing groundwater withdrawals (WD-CO_2 gw_) across the contiguous United States. (b) Total area-normalized county-level groundwater withdrawal DIC fluxes (WD-DIC_gw_) across the contiguous United States. (c) Total area-normalized county-level surface water withdrawal DIC fluxes (WD-DIC_sw_) across the contiguous United States. Scales represent the quintile groups. (d) Water use sector that makes the largest contribution to the gross total withdrawal DIC flux (WD-DIC_total_) for each county across the contiguous United States. Linework created using the “usmap” package in R.^48^

Median groundwater and surface water DIC concentrations (DIC_gw_ and DIC_sw_) across the US between 01.01.2010 and 31.12.2020 were modeled (using THINCARB) to be 48.2 and 29.7 mg C L^–1^, respectively. County-level surface water and groundwater DIC concentrations used within flux calculations and the corresponding method of determination are reported in Supplementary Data 1 and Figure S2. Gross median national-level fresh groundwater and surface water withdrawal DIC fluxes (WD-DIC_gw_ and WD-DIC_sw_) across the US were 6.9 (5.3–8.8) and 8.2 (6.7–9.9) Tg C yr^–1^, respectively (Table 1), with values in parentheses representing lower and upper estimates (Section 2.1). Irrigation and public supply withdrawals contribute 92% of the total WD-DIC_gw_ flux, and irrigation and thermoelectric withdrawals contribute 81% of the national-level WD-DIC_sw_ flux (Figure 1a). Counties with the largest area-normalized WD-DIC_gw_ and WD-DIC_sw_ fluxes were concentrated within the states of Nebraska (NE), Florida (FL), and California (CA; Figure 1b), and Montana (MT) and Wyoming (WY; Figure 1c), respectively. The water use sector making the largest contribution to total gross WD-DIC fluxes (WD-DI*C*_total_; the sum of WD-DIC_gw_ and WD-DIC_sw_) for each county across the US is shown in Figure 1d, with the irrigation and public supply sectors being the largest contributors to counties across the western and eastern regions of the country, respectively.

The national-level WD-DIC_gw_ flux (5.3–8.8 Tg C yr^–1^) was estimated to be 7–12 times larger than the median estimate of the US subterranean groundwater discharge DIC flux to the ocean (Table 1). The national-level WD-DIC_sw_ flux (6.7–9.9 Tg C yr^–1^) was equivalent to 7.9–11.6% of the outgassing of CO_2_ by rivers and lakes and 23.1–34.1% of the DIC exported to the oceans by rivers, making it equivalent to 5.9–8.7% of the total surface water DIC flux (Table 1). The gross national level WD-DI*C*_total_ flux (12–18.7 Tg C yr^–1^) was equivalent to 40.4–63.0% of the total discharge of DIC to the oceans from fresh groundwater and rivers across the US (Table 1).

#### 3.1.2 Net Dissolved Inorganic Carbon Fluxes Associated with Withdrawals of Fresh Water

**Figure 2:**
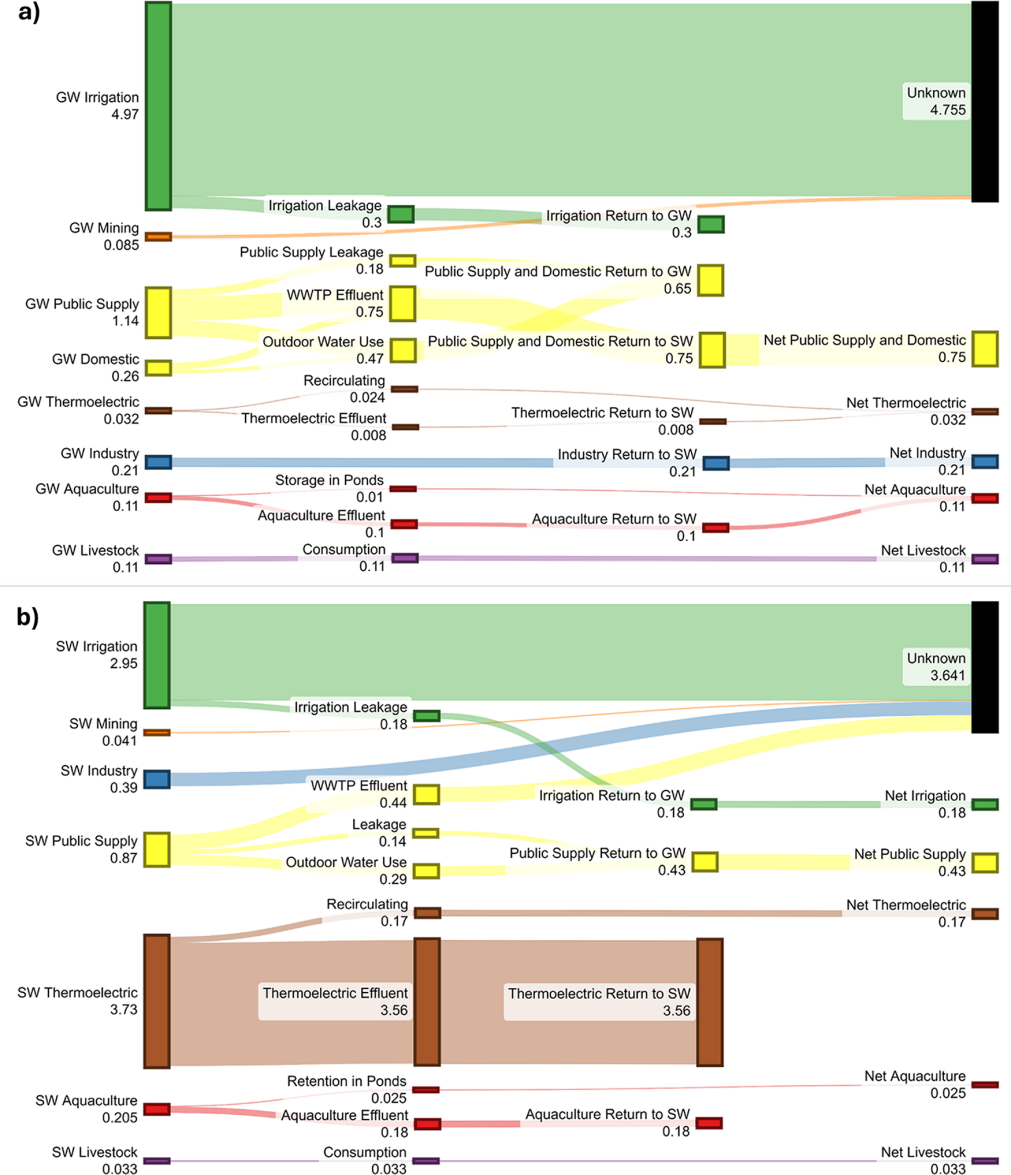
Median gross and net freshwater withdrawal DIC fluxes for each major water use sector across the United States. (a) Sankey diagram showing median gross and net freshwater withdrawal DIC fluxes for each major water use sector across the United States due to groundwater withdrawals. (b) Sankey diagram showing median gross and net freshwater withdrawal DIC fluxes for each major water use sector across the United States due to surface water withdrawals.

Net national-level WD-DIC fluxes that could be estimated in this research are summarized in Figure 2. It was estimated that 0.18 Tg C yr^–1^ and 0.30 Tg C yr^–1^ of the irrigation WD-DIC_sw_ and WD-DIC_gw_ fluxes could be returned to groundwater via leakage during irrigation conveyance, respectively. Determining the fate of DIC once both surface waters and groundwaters are used for irrigation is beyond the scope of this study (Section 3.2). Thermoelectric plants utilizing water-recirculating technologies result in net WD-DIC_gw_ and WD-DIC_sw_ fluxes of 0.024 Tg C yr^–1^ and 0.17 Tg C yr^–1^, respectively. The return of withdrawals from once-through cooling plants to surface water environments via effluents was estimated to cause net WD-DIC_gw_ and WD-DIC_sw_ fluxes of 0.008 and −0.008 Tg C yr^–1^, respectively. The reduced solubility of CO_2_ within once-through cooling plant effluents due to their elevated temperatures was estimated to cause the degassing of 0.35 Tg of CO_2_ yr^–1^ (Supplementary Note 3).

The return of WD-DIC_sw_ to groundwater due to leakage from public supply distribution pipes and outdoor water use at domestic residences resulted in a net public supply WD-DIC_sw_ flux of 0.43 Tg C yr^–1^. Approximately 95% of the remaining public supply and domestic WD-DIC_gw_ and WD-DIC_sw_ fluxes will be returned to wastewater treatment plants and subsequently be released into a surface water environment,^47^ resulting in a combined net public supply and domestic WD-DIC_gw_ flux of 0.75 Tg C yr^–1^.

The return of groundwater used within the industrial sector to surface water environments results in a net industry WD-DIC_gw_ flux of 0.21 Tg C yr^–1^. The consumption of water for livestock results in net WD-DIC_sw_ and WD-DIC_gw_ fluxes of 0.033 and 0.11 Tg C yr^–1^, respectively. The storage of fresh water within aquaculture ponds was estimated to temporarily retain 0.010 and 0.025 Tg C yr^–1^ from groundwater and surface waters, respectively. In addition to the storage of DIC in ponds, the return of water exclusively to surface water environments after aquacultural use results in a total net aquaculture WD-DIC_gw_ flux of 0.11 Tg C yr^–1^.

### 3.2 Degassing Groundwater Withdrawal CO_2_ Emissions

**Figure 3:**
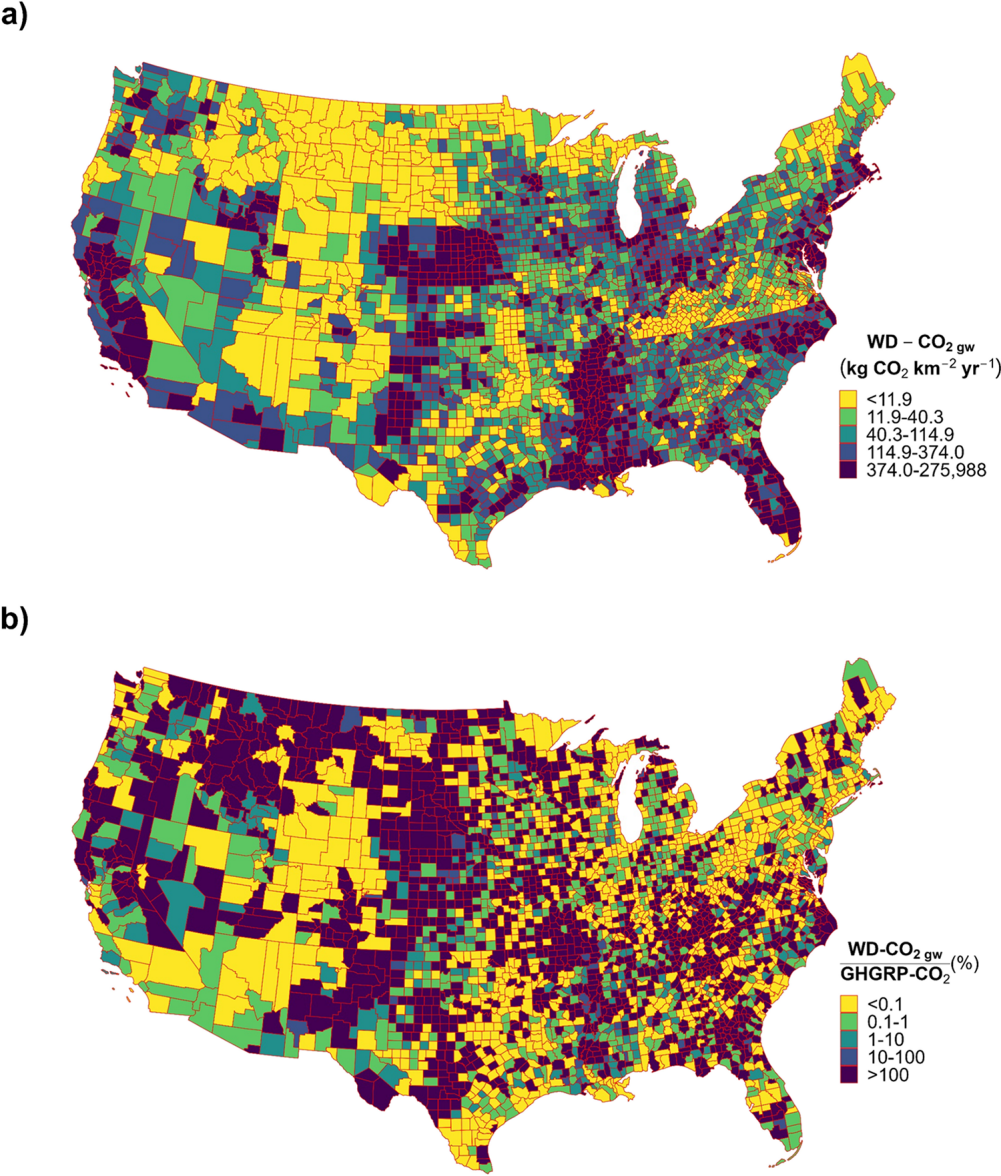
Emissions of carbon dioxide from degassing groundwater withdrawals across the United States. (a) Total area-normalized county-level emission of carbon dioxide due to groundwater withdrawals (WD-CO_2 gw_) across each county of the contiguous United States. (b) Percentage equivalence of carbon dioxide emissions due to degassing groundwater withdrawals (WD-CO_2 gw_), to the carbon dioxide emissions from the facilities required to report to the Greenhouse Gas Reporting Program (GHGRP-CO_2_), for each county across the contiguous United States. GHGRP-CO_2_ data was sourced from the USEPA.^45^ Linework created using the “usmap” package in R.^48^

Through the use of the THINCARB model, the median excess CO_2_ partial pressure of groundwater (EpCO_2_) across the US was estimated to be 29.2 (unitless), with 97% of samples being supersaturated relative to the atmosphere (EpCO_2_ > 1). The median national-level excess CO_2_ concentration of groundwater (E[CO_2 gw-atrm_]) was estimated to be 13.7 mg of CO_2_ L^–1^. Modeled EpCO_2_ values and calculated E[CO_2 gw-atm_] concentrations for all groundwater sites are reported in Supplementary Data 1. The national-level emission of CO_2_ due to the degassing of CO_2_ supersaturated groundwater withdrawals (WD-CO_2 gw_) across the US was estimated to be 3.6 (2.2–5.5) Tg CO_2_ yr^–1^ (Table 2), with irrigation and public supply withdrawals contributing 93% of this total (Figure 1a). Counties with the largest area-normalized WD-CO_2 gw_ fluxes were generally concentrated within the states of Nebraska (NE) and North Carolina (NC; Figure 3a).

**Table 2:** National-Level CO_2_ Emissions Associated with the Degassing of Withdrawn Groundwaters (WD-CO_2gw_), Expressed As a Percentage, Compared to Other Major National-Level CO_2_ Sources across the United States

flux (Tg CO_2_ yr^–1^)	outgassing by rivers and lakes (313)^a^	facility emissions (GHGRP-CO_2_) (2640)^b^	liming practices (3.8)^c^	electricity use for pumping irrigation groundwater (10.7)^d^	degassing of CO_2_ from groundwater irrigation withdrawals (1.43)^e^	electricity generation for drinking water system operation (26.5)^f^
**national-level total**						
WD-CO_2gw_ (2.2–5.5)	0.7–1.8	0.08–0.2				
**national-level irrigation**						
WD-CO_2gw_ (1.6–3.9)			42.1–102.6	14.9–36.4	112–273	
**national-level public supply & domestic**						
WD-CO_2gw_ (0.5–1.1)						1.8–4.1

a Butman, Stackpoole, Stets, McDonald, Clow and Striegl.^3^

b USEPA.^45^

c USEPA.^42^

d Driscoll, Conant, Marston, Choi, and Mueller.^43^

e Qin, Duan, Zou, Chen, Huang, and Rosa.^25^

f Zib, Byrne, Marston, and Chini.^44^

The national-level WD-CO_2 gw_ flux (2.5–5.5 Tg CO_2_ yr^–1^) was estimated to be equivalent to between 0.7 and 1.8% of the CO_2_ outgassed by rivers and lakes and 0.08–0.2% of total CO_2_ emissions from major directly emitting facilities required to report to the US EPA’s Greenhouse Gas Reporting Program (GHGRP-CO_2_; Table 2). The national-level WD-CO_2 gw_ flux due to irrigation withdrawals (1.6–3.9 Tg CO_2_ yr^–1^) was equivalent to 42.1–102.6% of the CO_2_ emissions associated with the country’s liming practices and between 14.9 and 36.4% of the CO_2_ emissions associated with electricity generation for pumping groundwater for irrigation use. The national-level WD-CO_2 gw_ flux due to public supply and self-supplied domestic withdrawals (0.5–1.1 Tg CO_2_ yr^–1^) was equivalent to between 1.8 and 4.1% of the CO_2_ emissions associated with electricity generation for the operation of US drinking water systems (Table 2). Approximately 45% of all US counties (1,401) were estimated to have median WD-CO_2 gw_ fluxes that exceeded county-level GHGRP-CO_2_ emissions, with these counties concentrated in the states of Montana, South Dakota (SD), Nebraska, and Idaho (Figure 3b).

## 4 Discussion

### 4.1 Anthropogenic Withdrawals of Fresh Water Perturb the Lateral Transport of DIC

This research provides the first insights into how anthropogenic withdrawals of fresh water across the US may act as an important DIC retention mechanism, delaying the delivery of DIC to the ocean via fresh subterranean groundwater discharge and surface water export. The magnitude of the country’s gross surface water and groundwater withdrawal DIC fluxes (WD-DIC_sw_ and WD-DIC_gw_), in comparison to other DIC fluxes to the oceans, suggests that withdrawals may cause important perturbations to overall national-level DIC cycling (Table 1). Many overall C budgets are determined using a mass balance approach. These findings therefore emphasize the importance of incorporating WD-DIC fluxes into national-scale C cycling budgets as a way of more accurately determining other budget components.^3,11,16,18^ Uncertainties associated with gross WD-DIC fluxes, largely due to the scarcity of measured DIC concentration data (Supplementary Note 1), should be reduced as more temporally and spatially resolved water use and DIC concentration data become available.^49^

### 4.2 Sector Dependent Controls on Net DIC Fluxes

It is important to note that estimating gross WD-DIC fluxes provides a necessary first step in understanding the net impact of freshwater withdrawals upon DIC cycling across the US. Subsequent to freshwater withdrawal, a vast range of interlinked hydrological, biological, and chemical processes will modify the amount of DIC that is either retained or returned to fresh waters. This means that gross WD-DIC flux estimates often exceed those of their net WD-DIC flux counterparts. Comparing gross WD-DIC fluxes with other major DIC fluxes is intended to assess the potential maximum magnitude and importance of WD-DIC fluxes in the broader context of freshwater DIC cycling.

The retention of DIC through the consumption and storage of water varies across the country and between different water use sectors.^50^ The capacity for recirculating thermoelectric plants to temporarily store DIC is small compared to those of other naturally occurring mechanisms that remove DIC from fresh waters, such as riverine export of DIC to the oceans (Figure 2; Table 1). However, this capacity may increase in the future given the predicted transition to recirculating technologies across the US.^51^ The temporary storage of water within thermoelectric plants, aquaculture ponds, and both irrigation and municipal water towers may impact both DIC burial and CO_2_ emissions.^12^ The storage and consumption of water within industrial and mining water use sectors are largely unknown and complex to estimate,^52−54^ contributing to unresolved net WD-DIC flux estimates for these sectors.

Return flows of withdrawn water can also redistribute water and associated DIC between groundwater and surface water environments.^55,56^ Industrial, thermoelectric, public supply, and aquaculture sectors return both groundwater and surface water almost exclusively to surface water environments via effluents, resulting in the net removal of DIC from groundwater (Figure 2a). Conversely, the leakage of water from main distribution pipes and return of water during outdoor water use can return biologically important nutrients to subsurface environments,^32,57^ thus resulting in the net removal of DIC from surface waters (Figure 2b). The use of water for irrigation and mining will also result in the complex and localized movement of water and associated DIC between groundwater, surface water, and atmospheric environments.^55,58−61^ However, there are no comprehensive national-level data sets disclosing the volume of water that is retained and returned to each environment. For example, the consumptive use of surface water for irrigation across the US was modeled to decrease fresh surface water discharge to the ocean by 4.2%.^59^ A lack of data relating to whether this consumption was due to evaporative loss or reallocation to groundwater, as well as any associated C speciation changes, hinders the determination of net irrigation WD-DIC_sw_ flux. Despite this, the (4.2%) decrease in surface water export can be used as a means of validating the magnitude of our irrigation WD-DIC_sw_ flux. Applying a proportional decrease in the reported river DIC export flux (29 Tg C yr^–1^)^3^ would result in a retention flux of 1.3 Tg C yr^–1^ due to fresh surface water withdrawals for irrigation, which is around 44% of our gross WD-DIC_sw_ flux estimate (2.95 Tg C yr^–1^).

The use of water for irrigation, mining, industrial, and public supply sectors will also lead to changes in DIC speciation and concentration. For example, freshwater withdrawals can cause increased evaporation,^59^ which in turn may increase DIC concentrations within the remaining water. However, this may also occur in tandem with the precipitation of carbonate minerals within soils, CO_2_ emissions, and the utilization of DIC for primary production, which can act to decrease DIC concentrations and the amount of DIC that can be leached to groundwaters or transported in runoff to surface waters.^62^ However, data relating to mechanisms controlling these speciation and concentration changes remain spatially limited, and alongside the lack of comprehensive data regarding the impact of freshwater withdrawals upon the US water balance, net national-level WD-DIC_gw_ fluxes for irrigation and mining sectors (Figure 2a), and net WD-DIC_sw_ fluxes for irrigation, industrial, mining and public supply sectors remain unknown (Figure 2b). Research priorities and data needed to resolve these issues are highlighted in Section 4.4.

Our research has also estimated the impact of increased thermoelectric plant effluent temperatures on national-level CO_2_ emissions to the atmosphere (Supporting Information Note 3). More localized assessments could also be conducted to identify hotspots of these emissions, particularly across eastern regions of the country, where once-through technologies responsible for thermal pollution are more common.^63^ Estimating CO_2_ emissions across heavily thermally polluted river systems worldwide will be necessary to understand the impact of thermal pollution upon global CO_2_ degassing.^64^

### 4.3 Degassing of CO_2_ from Groundwater Withdrawals

The degassing of CO_2_ supersaturated groundwater withdrawals across the US is known to contribute to the country’s atmospheric CO_2_ emissions.^21,23,25^ The research reported here uses a more robust methodology to estimate the subnational and sectoral contributions to a national-level CO_2_ emission of 3.6 Tg CO_2_ yr^–1^ (Figure 1a). This value is 112% larger than the 1.7 Tg CO_2_ yr^–1^ previously reported,^21^ primarily due to the use of total groundwater withdrawal volumes within our calculations, as opposed to the lower volumes that represent groundwater depletion, or net withdrawals used by Wood and Hyndman.^21^ Although our research uses larger (gross) withdrawal volumes for estimating WD-CO_2 gw_ emissions, as opposed to net (depletion) withdrawal volumes, the excess groundwater CO_2_ concentrations (E[CO_2 gw-atm_]) determined and used within our research are on average lower than those adopted in previous work.^21^ We believe that the approach reported in the current paper is conceptually more representative of the amount of CO_2_ degassed, as withdrawn groundwater will degas more rapidly than the time it takes for it to be returned to an aquifer,^39,40^ and that the use of THINCARB has modeled more spatially resolved and accurate E[CO_2 gw-atm_] concentrations across the US than in previous research. The use of lab-measured pH values within calculations, due to a lack of reported field pH values, may lead to an underestimate of CO_2_ emissions from groundwater.^40^ The use of both in-field and lab-measured pH values in the determination of WD-CO_2 gw_ emissions is therefore discussed in Supplementary Note 1.

Ninety-six percent of the 1401 counties that have WD-CO_2 gw_ emissions exceeding those from major emitting facilities (Figure 3b) have no emissions reported as part of the GHGRP,^45^ which is assumed to largely reflect the fact that any emissions from facilities within those counties are below the reporting threshold.^46^ Despite this, our identification of regions where WD-CO_2 gw_ emissions are important in relation to other major CO_2_ emission sources (Figure 3b) suggests that these emissions should be included within regional and local-scale C budgets, C footprint assessments, and Net Zero efforts by the US water supply sector.^65^

Previous work has generally focused on quantifying the CO_2_ emissions associated with degassing groundwater withdrawals for irrigation use.^23−25^ A more comprehensive assessment of the sectoral withdrawals that can contribute to the total withdrawal of CO_2_ emissions has been made in our current research, with observed sectoral differences in WD-CO_2 gw_ emissions driven by the contrasting dependence of each water use sector on groundwater withdrawals. Although irrigation groundwater withdrawals make a dominant contribution to total national-level WD-CO_2 gw_ emissions, neglecting groundwater withdrawals from other water use sectors would cause a 27% underestimate of WD-CO_2 gw_ emissions (Figure 1a). With the volume of groundwater withdrawals anticipated to increase across many regions of the US,^66^ WD-CO_2 gw_ emissions are likely to persist or even increase into the future. While beyond the scope of this research, the E[CO_2 gw-atm_] data set and methodology presented in the current paper should facilitate a more detailed investigation into the mechanisms controlling E[CO_2 gw-atm_] concentrations and thus WD-CO_2 gw_ emissions. This is likely to include consideration of land use^67,68^ and hydrogeological setting^69^ (e.g., Figure S3). An improved understanding of these mechanisms would then support more sustainable groundwater management strategies, not only for the purpose of conserving fresh groundwater resources but also for the regulation of atmospheric CO_2_ emissions.

### 4.4 Future Priorities for Estimating Net DIC Fluxes

Estimating net US irrigation WD-DIC fluxes is complex and beyond the scope of this research. As the largest sector contributing to the gross national-level WD-DIC flux, estimating the net impact of irrigation withdrawals on DIC cycling may impact the determination of other sectoral net WD-DIC fluxes and thus warrants additional research. This will require comprehensive country-wide data sets detailing the amount of water withdrawn that is subsequently used for irrigation, as any spare water withdrawn will be stored within reservoirs.^70^ Data disclosing the varying proportion of water returned to either surface waters or groundwaters post irrigation,^28^ as well as the physiochemical changes associated with this redistribution of water will also be needed.^14^ Modeling the fate of DIC both during and post irrigation will also be required to determine how much DIC is taken up by crops, precipitated as carbonate within soils, or degassed as CO_2_. This will require a substantial range of input data sets. For example, the amount of DIC degassed as CO_2_ both during and post irrigation may require data relating to irrigation system type (flood, sprinkler, or drip), irrigation efficiency, return flows, as well as soil and crop type.^71−74^ While research has estimated the energy-derived CO_2_ emissions from surface water pumping and running of surface irrigation systems across the US,^25^ understanding the impact of different irrigation systems on CO_2_ degassing from both withdrawn surface waters and groundwaters across the country remains an important area of research.^71^

Similarly, a lack of comprehensive national-level data detailing potable water treatment and in-pipe processes that may affect DIC speciation and retention, such as pH adjustment and the precipitation of carbonates within potable water distribution pipes,^75^ limits our ability to accurately determine a net WD-DIC_sw_ flux at this time (Figure 2b). We estimate the input of DIC via public supply return flows (WWTP effluent) to be 2.9 Tg C yr^–1^ (Supplementary Note 2), a flux that exceeds the retention capacity provided by withdrawals, with combined WD-DIC_gw_ and WD-DIC_sw_ fluxes (1.2 Tg C yr^–1^) equivalent to 41% of the WWTP effluent DIC flux. Despite this exceedance, future research should continue to resolve the retention of DIC within the water distribution system and the degree to which freshwater withdrawals can moderate the downstream export of potentially environmentally disruptive DIC inputs from municipal wastewater effluents.^76^

Although withdrawals for industrial, aquaculture, and mining water use sectors are minor on a large (global and national) spatial scale, when compared to irrigation and public supply sectors, they can make major contributions to overall freshwater withdrawals on more localized scales. In addition, these sectors often withdraw water within environmentally sensitive locations,^52^ meaning they may have an important impact on overall freshwater nutrient cycling within an area. Data detailing the proportion of water withdrawn for industrial, aquaculture, and mining water use sectors that is stored and returned to surface water and groundwater environments,^50,58^ as well as any associated DIC concentration changes, are currently limited on a national scale. Annual county-level WD-DIC fluxes neglect to account for both the seasonality and the spatial heterogeneity in both freshwater use and DIC concentrations.^77,78^ A lack of data has also prevented the use of sector-specific DIC concentrations within flux calculations. Should calls for more widespread and regular in situ monitoring of freshwater quality determinants (including pH, CO_2_, and DIC) be answered^11,79^ and more spatially and temporally resolved water use data released, uncertainties associated with gross and net sectoral WD-DIC fluxes should also be reduced. With 10% of counties responsible for over 70% of total freshwater consumption across the country,^50^ efforts to determine net WD-DIC fluxes could be prioritized in these areas. While this work highlights the localized importance of WD-CO_2 gw_ emissions, future work must also determine to what extent human-induced groundwater withdrawal CO_2_ emissions affect the amount of CO_2_ degassing by natural discharge downstream.^40^

This paper considers freshwater withdrawals and reservoir creation, through the damming of surface waters, to be distinct processes capable of impacting freshwater DIC cycling.^12^ Fresh surface water withdrawal data used in this paper do not distinguish between withdrawal from reservoirs or other surface water bodies.^33^ However, approximately 15% of US dams are constructed for municipal and irrigation water supply purposes,^80^ and processes controlling DIC concentrations within the lentic environment of reservoirs often differ substantially from those within the wider lotic network of a river or stream.^81^ If future data allow differentiation between freshwater withdrawals from reservoirs versus other surface waters, it will be important that research more accurately constrains the specific controls exerted by reservoirs on DIC concentrations and thereby on withdrawal DIC fluxes from these freshwaters. Integrating the impacts of water supply reservoirs and fresh surface water and groundwater withdrawals upon DIC cycling across the contiguous US (e.g., ref (28)) is beyond the scope of this study, however constitutes an important piece of future research. Subsequent withdrawals of freshwater from these dammed areas can also lead to a release of atmospheric CO_2_ emissions due to an increase in drawdown area.^27,82^ Future work should integrate degassing groundwater withdrawal CO_2_ emissions with other water supply-related CO_2_ emissions, including those from drawdown, surface water withdrawal degassing, thermoelectric effluents, and aeration during irrigation.

### A Global Perspective on the Impacts of Water Withdrawals on Freshwater–Carbon Fluxes

While the research reported here has estimated the impacts of freshwater withdrawals on freshwater C fluxes across the contiguous US, withdrawals of fresh water are likely to perturb freshwater C fluxes globally. Using global net groundwater and surface water withdrawal volumes^83^ and adopting median US DIC_gw_ and DIC_sw_ concentrations determined in this study, we estimate net global WD-DIC_gw_ and WD-DIC_sw_ fluxes to be 12.4 and 35.0 Tg C yr^–1^, respectively. We estimate the global subterranean groundwater discharge (SGD) DIC flux (DIC _SGD_), using lower and upper fresh global SGD volume estimates^7^ and the median US DIC_gw_ concentration determined in this study, to be 269.6–770.3 Tg C yr^–1^. Net global WD-DIC_gw_ and WD-DIC_sw_ fluxes may therefore be equivalent to approximately 1.6–4.6% of the global DIC_SGD_ flux and 8.6% of global riverine DIC export,^6^ respectively. These coarse calculations highlight the potential importance of net freshwater withdrawal DIC fluxes, with respect to global freshwater DIC cycling. Using recent global groundwater withdrawal volumes (959 km^3^ yr^–1^, for the year 2017)^84^ and the median excess groundwater CO_2_ concentration estimated in this study (13.7 mg CO_2_ L^–1^), we estimate the global WD-CO_2 gw_ flux to be 13.1 Tg CO_2_ yr^–1^. This estimate is slightly lower than the upper depletion WD-CO_2 gw_ flux (9.7–13.5 Tg CO_2_ yr^–1^) made by Wood and Hyndman,^21^ an artifact of the simultaneously higher gross WD_gw_ value but lower E[CO_2 gw-atm_] concentration adopted in the research we report here. This estimate is much lower than the 36.7–110 Tg CO_2_ yr^–1^ estimated by Macpherson^40^ due to the lower E[CO_2 gw-atm_] concentration adopted in this study. Emerging global data sets estimating sectoral water use and consumption should be used to resolve similar fluxes elsewhere around the world,^85^ with priority given to countries undertaking globally significant withdrawals of fresh water.

To conclude, fresh surface water and groundwater withdrawals across the US were estimated to result in gross dissolved inorganic carbon (DIC) retention fluxes of 6.7–9.9 and 5.3–8.8 Tg C yr^–1^, respectively. The degassing of CO_2_ supersaturated groundwater following withdrawal was estimated to emit 2.2–5.5 Tg of CO_2_ yr^–1^, 112% larger than previous estimates, with county-level CO_2_ emissions exceeding CO_2_ emissions from major emitting facilities across 45% of US counties. Future work should continue to resolve net US freshwater withdrawal DIC fluxes and CO_2_ emissions as more data becomes available. Results should then be integrated into wider carbon budget assessments and help inform more sustainable management of freshwater resources and carbon cycling.

## Supporting Information Available

Additional details regarding data quality assurance and methodologies used throughout the manuscript are detailed in Supporting Information document (PDF)
The dataset disclosing fresh groundwater and surface water DIC concentrations and excess CO_2_ concentrations of fresh groundwaters across the United States, including steps taken to calculate excess CO_2_ concentrations of fresh groundwaters are also detailed (XLSX)

## Notes

All data used within this research are publicly available. Volumetric rate data of freshwater withdrawals were sourced from the USGS^33^ and DIC concentrations were modeled using data from the Water Quality Portal.^34^ Data surrounding processes affecting DIC returns were available from a variety of sources, see Supporting Information. Linework on map figures were created using the “usmap” package in R.^48^
