# Supplementary material for: Anthropogenic Water Withdrawals Modify Freshwater Inorganic Carbon Fluxes across the United States
Source: Environ Sci Technol. 2025 Feb 17;59(8):3949–60. doi: 10.1021/acs.est.4c09426 (PMC11883805; doi:10.1021/acs.est.4c09426)
Supplement: Supplementary file 1 — es4c09426_si_001.pdf [file es4c09426_si_001.pdf]

Supplementary Information

Anthropogenic Water Withdrawals Modify Freshwater Inorganic Carbon Fluxes Across the United States

Elizabeth M. Flint[1,2*], Matthew J. Ascott[1], Daren C. Gooddy[1,2,3], Mason O. Stahl[4], Ben W.J. Surridge[2]

[1] *British Geological Survey, Maclean Building, Crowmarsh, Oxfordshire, United Kingdom, OX10 8BB*

[2] *Lancaster Environment Centre, Lancaster University, Lancaster, United Kingdom, LA1 4YQ*

[3] *UK Centre for Ecology and Hydrology, Maclean Building, Crowmarsh, Oxfordshire, United Kingdom, OX10 8BB*

[4] *Department of Geosciences, Union College, Schenectady, New York, United States, 12308*

*Corresponding author: Elizabeth May Flint (efli1@bgs.ac.uk)

19 pages

5 figures

3 tables

4 supplementary notes

Supplementary Note 1. Determination of Fresh Groundwater and Surface Water DIC Concentrations and Estimation of Withdrawal DIC Fluxes

The most recent release of water use data from the USGS discloses annual total water use for each defined major water use sector across each county of the United States [1]. As a result, annual freshwater withdrawal DIC fluxes were estimated, where possible, using annually averaged (median) county-level freshwater DIC concentration values. Due to limited availability of a county-level freshwater DIC concentration value, 1,621 and 2,233 counties had to adopt state-level median fresh groundwater and surface water DIC concentrations within flux calculations, respectively (Figure S1 and S2). Freshwater DIC concentrations are spatially heterogeneous [2]. The state-level aggregation of DIC concentration data within county-level flux calculations will have therefore given rise to an increase in the uncertainty associated with flux estimates, as expressed by lower and upper estimates made using 25th and 75th percentile DIC concentrations. It should be noted that even the use of an annual median county-level DIC concentration neglects the inherent local and seasonal variability in freshwater DIC concentrations [2, 3]. Despite this, the use of a single state or county-level median DIC concentration provides an appropriate starting point for estimating these fluxes on a spatial scale as large as the contiguous United States. It should be noted that median fresh groundwater and surface water DIC concentrations were adopted in flux calculations due to the skewed distribution of DIC concentration data (Figure S4).

Estimating freshwater withdrawal DIC fluxes on higher resolution temporal (e.g. monthly) and spatial (e.g. for individual intakes and wells) scales, initially focusing on WD-DIC flux hotspots identified in this work, will facilitate a more accurate assessment of freshwater withdrawal DIC fluxes. It would also provide more insight into heterogeneity of anthropogenic influences, natural controls and ecological impacts of these fluxes, which could feed into the development of more tailored and effective water management decisions. Increasing the spatial and temporal resolution of freshwater withdrawal DIC flux estimates should therefore be a future research priority, as the required data becomes increasingly available. For example, data disclosing freshwater withdrawal volumes and DIC concentrations associated with individual US reservoirs are currently lacking, with reservoir withdrawals currently making an unknown contribution to surface water withdrawals [1]. Reservoirs for water supply purposes are widespread across the US [4], with approximately 15% of US dams being constructed for municipal and irrigation water supply purposes [4, 5]. The physical, biological and chemical characteristics of individual reservoirs are highly variable, which in turn may have a substantial influence on the DIC concentrations found within them [6]. The contribution of withdrawals from individual reservoirs to overall US WD-DIC$_{sw}$ fluxes and downstream DIC export should therefore be determined in future research, assuming relevant data become available.

The workflow developed in this research for obtaining DIC_{gw} and DIC_{sw} concentrations is outlined in Figure S1. Measured DIC concentrations generally carry less uncertainty than calculated DIC concentrations, predominantly due to the sensitivity of calculations to the accuracy of a measured pH value (see below discussion on data quality). Although the use of directly measured freshwater DIC concentrations is therefore preferable, values are scant and only available for fresh surface waters within 79 US counties (Figures S1 and S2). Bridging this gap in freshwater DIC concentration data was done so through the subsequent use of a thermodynamic inorganic carbon model (THINCARB) and equilibrium calculations. Although there is likely to be some degree of bias in the selection of locations for water quality testing across the United States, the use of each of these methods produced a DIC concentration dataset with sites distributed across varying land uses and geologies (Figure S3). Where possible, modelled DIC_{sw} concentrations were validated against measured DIC_{sw} concentrations that had been determined during the same sampling activity (n = 2,961), with this approach concurrent with other literature [7]. The agreement between modelled and measured DIC_{sw} values was strong, with a regression slope close to one (Figure S5). Data required to calculate (pH, carbonate and bicarbonate) and model (pH, alkalinity, temperature, altitude and calcium) DIC were queried either directly using the Water Quality Portal's 'Advanced Search' tool or via the use of the 'dataRetrieval' package in R, within a date range of 01.01.2010 and 31.12.2020. Queries for alkalinity, bicarbonate and carbonate data were only made for groundwater (site type: well) or surface water (site type: stream, lake, reservoir or impoundment) samples that had been filtered, as this will have helped to calculate a more representative DIC concentration value. Although sampling protocol states to prevent agitation during water sampling, the potential for aeration with the atmosphere during the filtration of water samples may have resulted in an underestimation of $E[CO_{2\ gw\text{-}atm}]$ concentrations and thus WD-$CO_{2\ gw}$ emissions [8].

Field measured pH values (opposed to laboratory measured pH values) were preferably used within THINCARB modelling and DIC equilibrium equations, due to the potential for pH values to reduce between sample acquisition and laboratory analysis. 53% and <1% of groundwater and surface water pH values used within equilibrium calculations and 67% and 84% of groundwater and surface water pH values used within THINCARB model inputs were explicitly reported as being measured in the field, respectively (Table S1).Where a site had both reported field and laboratory pH values, the impact of using laboratory measured pH values upon DIC_{sw}, DIC_{gw} and $E[CO_{2\ gw\text{-}atm}]$ concentrations (opposed those measured in the field) was investigated. Using laboratory pH values, the median output of DIC_{gw} and DIC_{sw} concentrations across the country were -7% and -1% of the DIC_{gw} and DIC_{sw} concentrations determined using field measured pH values, respectively, and therefore within reasonable uncertainty bounds (i.e. ±10%). In

contrast, it is known that a lack of accurately reported field pH values may underestimate WD-$CO_{2\ gw}$ fluxes [9]. The median difference between calculating E[$CO_{2\ gw\text{-}atm}$] concentrations using lab versus field pH values was -70%. Despite this, the majority (67%) of groundwater pH values used within this analysis were measured in the field (in-situ). This difference will therefore have likely resulted in a slight underestimate of WD-$CO_{2\ gw}$ emissions. Ultimately, this work supports calls for more widespread and regular in-situ monitoring of freshwater quality determinants, including pH, CO_2 and DIC [10, 11].

The inherent quality of data from the Water Quality Portal has also been investigated (Table S1). It was found that the majority of water quality data points used in this analysis have a final status of 'accepted', opposed to 'preliminary' (Table S1), suggesting that data used within this work has undergone quality assurance and quality control checks (e.g. acceptable ion balance), and is therefore of reliable and publishable quality. The status of alkalinity, carbonate and bicarbonate results used within this analysis is shown in more detail within Table S1. The method that was used to calculate each county-level DIC concentration, the number of water quality samples used to deduce the median DIC value for each county and the steps involved to calculate DIC and EpCO_2 values is detailed in Supplementary Dataset 1.

Table S1. Table of parameter codes used to obtain parameter inputs for subsequent use in THINCARB modelling and equilibrium DIC calculations. The percentage of returned water quality measurements that have been accepted and percentage of pH values measured in-situ that were used within the analysis are also detailed.

Parameter	Parameter Code	% of Values Accepted	% pH Values Measured in Field
**Groundwater (Wells)**			
Equilibrium Calculations			
pH	00400, 00403, 00408	-	53
Carbonate and bicarbonate	00452, 29807, 29808, 29809, 63788, 00453, 29804, 29805, 29806, 63786	90	-
THINCARB			
pH	00400, 00403, 00408	-	67
Calcium	00915, 91051	-	-
Alkalinity	00418, 00421, 29801, 29802, 39036, 39086, 39087	74	-
**Surface Water (Streams, Lakes, Reservoirs and Impoundments)**			
Measured DIC			
DIC	00691	86	-
Equilibrium Calculations			
pH	00400, 00403, 00408	-	<1
Carbonate and bicarbonate	00452, 29807, 29808, 29809, 63788, 00453, 29804, 29805, 29806, 63786	99.9	-
THINCARB			
pH	00400, 00403, 00408	-	84
Calcium	00915, 91051	-	-
Alkalinity	00418, 00421, 29801, 29802, 39036, 39086, 39087	93	-

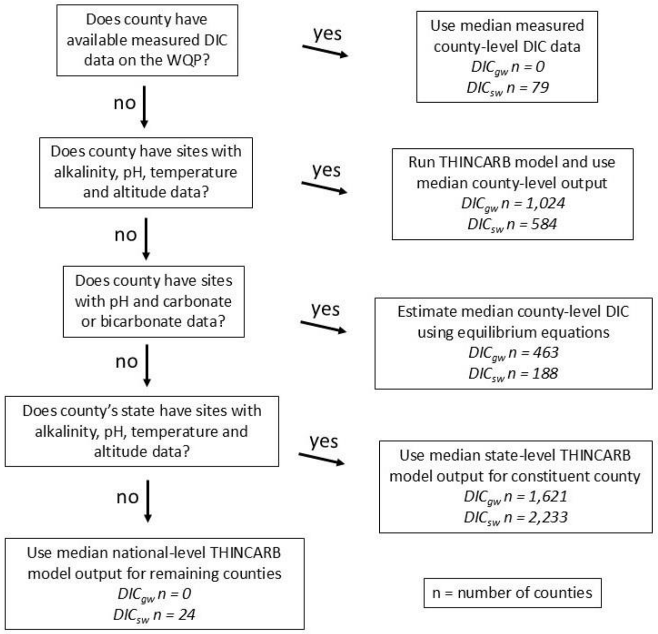

Figure S1. Schematic outlining the methodology used to determine county-level freshwater dissolved inorganic carbon concentrations across the United States. Dissolved inorganic carbon (DIC) concentrations are seldom measured and reported on the Water Quality Portal (WQP). To expand the DIC concentration dataset, this research made use of a thermodynamic inorganic carbon model (THINCARB) and equilibrium calculations. The number of counties (n) that made use of each method to obtain groundwater and surface water DIC concentrations (DIC_{gw} and DIC_{sw}) are detailed.

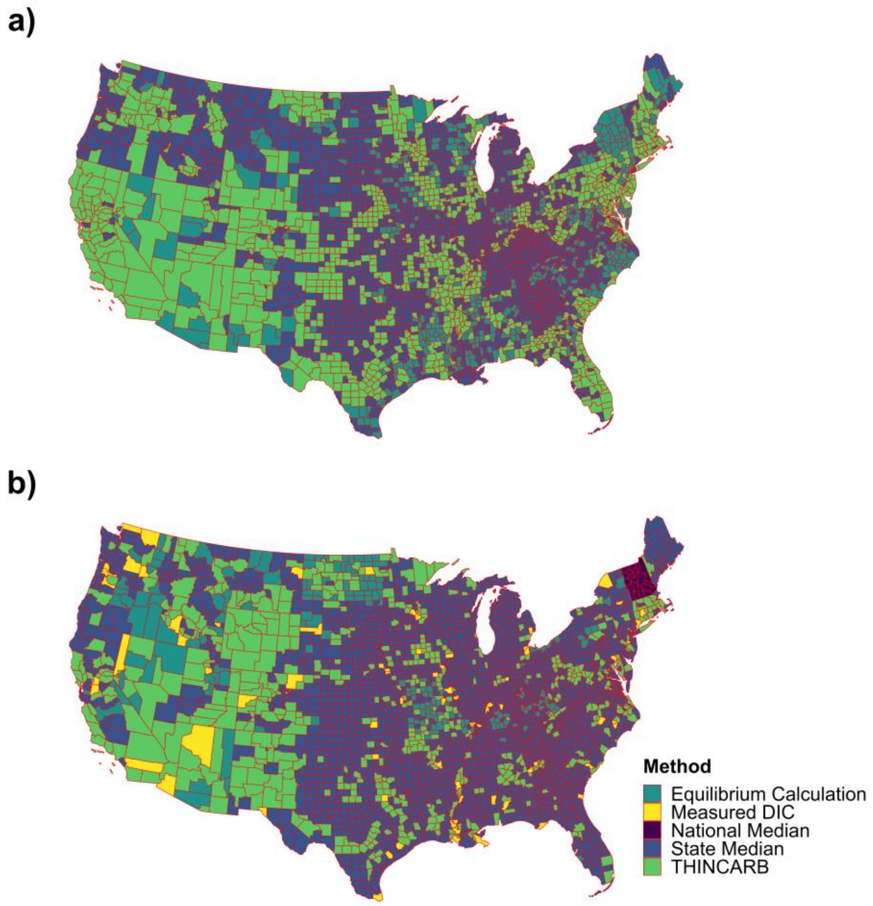

Figure S2. Methodology used to determine fresh water DIC concentrations for each county across the United States. a) Methodology used to determine fresh groundwater DIC concentrations. **b)** Methodology used to determine fresh surface water DIC concentrations. Linework created using the 'usmap' package in R [12].

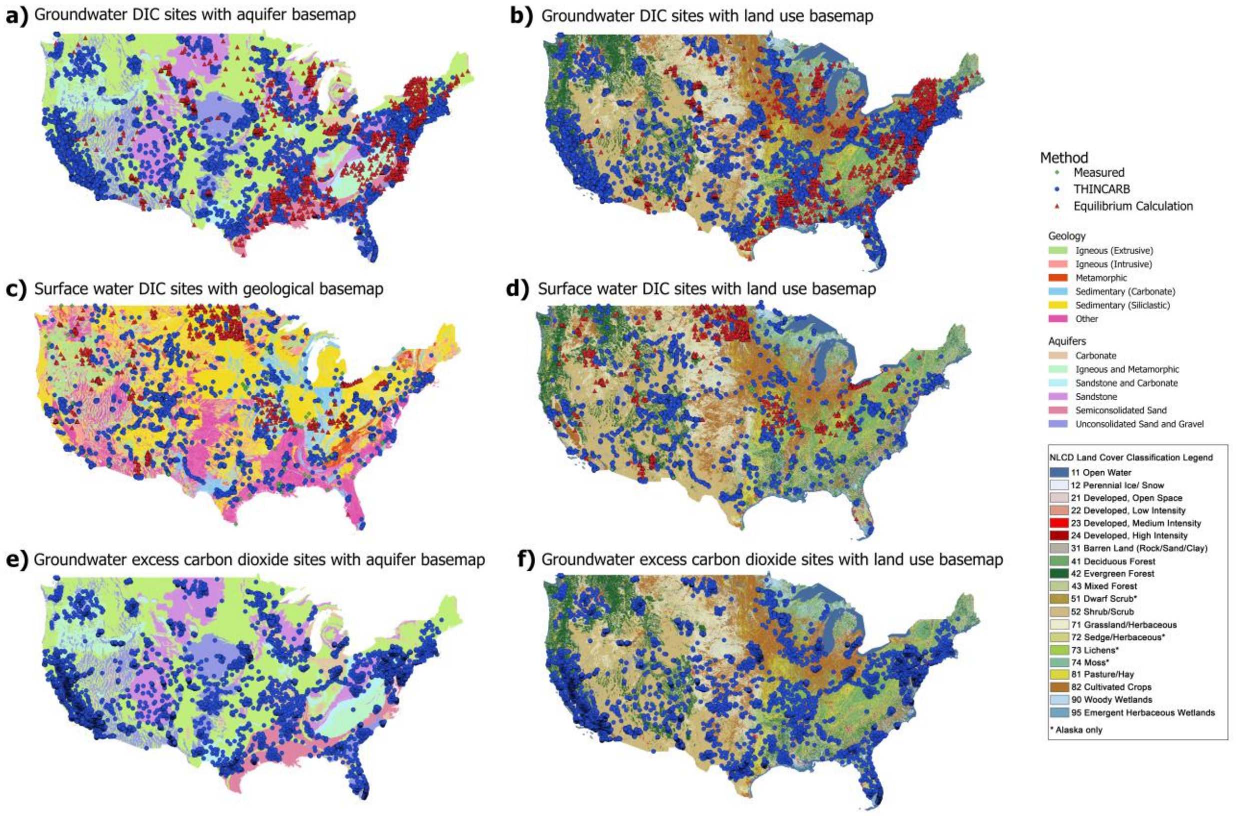

Figure S3. Maps showing the distribution of freshwater DIC and E[$CO_{2\ gw\text{-}atm}$] concentration data points estimated using different methods across the United States. Maps showing the distribution of sites where fresh groundwater DIC concentrations were estimated, in the context of **a)** principal aquifers [13] and **b)** land uses [14] across the United States. Maps showing the distribution of sites where fresh surface water DIC concentrations were estimated, in the context of **c)** geology [15] and **d)** land use [14] across the United States. Maps showing the distribution of sites where fresh groundwater E[$CO_{2\ gw\text{-}atm}$] concentrations were estimated, in the context of **e)** principal aquifers [13] and **b)** land uses [14] across the United States.

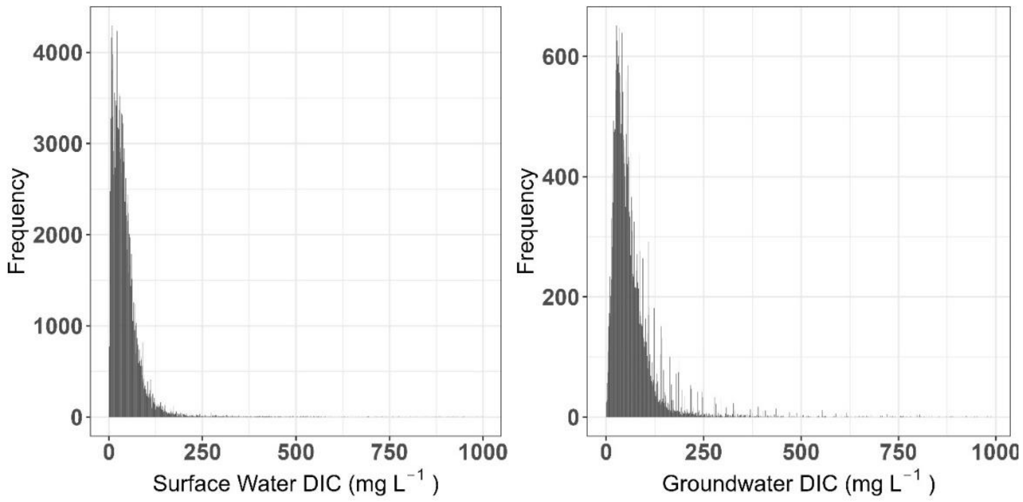

Figure S4. Histograms show the distribution of fresh surface water and groundwater dissolved inorganic carbon concentration data points used within this analysis. The non-normal distribution (right skewness) of measured and modelled DIC concentration data provided justification for adopting median county-level DIC concentrations within DIC flux calculations. These plots support results of a Kolmogorov-Smirnov test (p-value < 0.05), that also indicates the non-normal distribution of DIC concentrations.

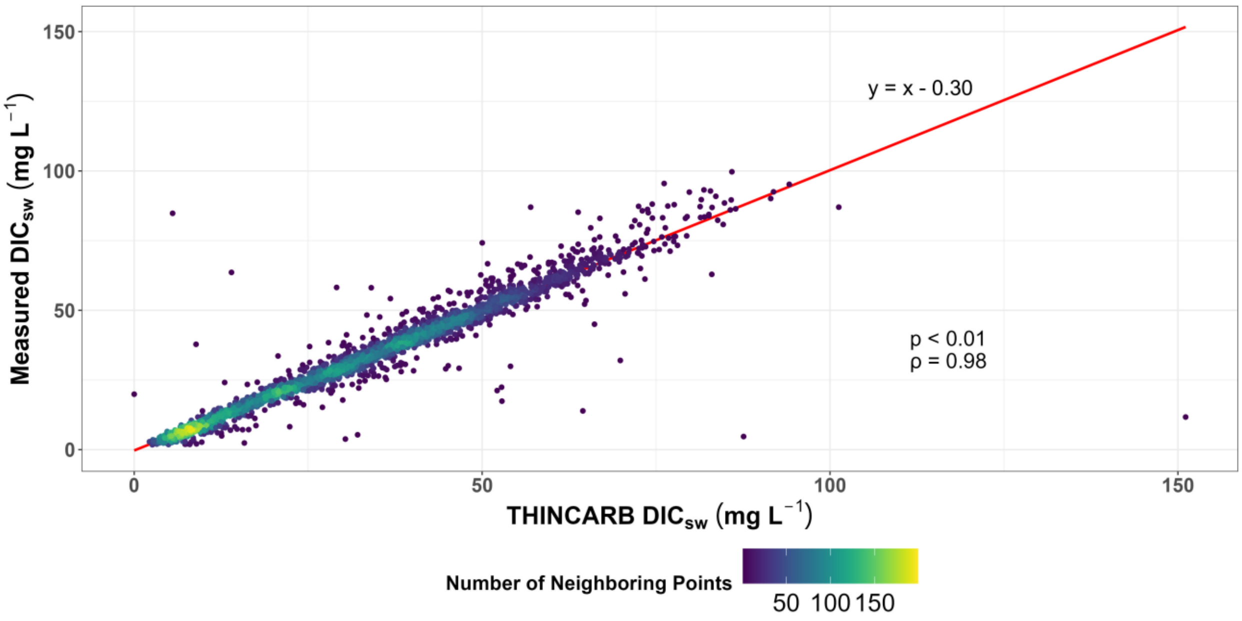

Figure S5. Measured surface water DIC concentrations (WQP, 2023) compared to those modelled using THINCARB. The regression line is shown in red, with a Spearman's correlation coefficient (ρ) value of 0.98 and p-value < 0.01.

Supplementary Note 2. Estimating Net Freshwater Withdrawal DIC Fluxes for Major Water Use Sectors Across the Contiguous United States

The following assumptions and methodologies were adopted to make initial net freshwater withdrawal DIC fluxes (Figure 2). These are defined as the fluxes of DIC that are removed from downstream transport on timescales relevant to overall C cycling, due to either groundwater or surface water withdrawals, having accounted for consumption, return flows and speciation changes.

Irrigation

Some previous work has assumed that once fresh water has been used (for all water use sectors, including irrigation) it will be returned to downstream surface water bodies [16, 17], whilst other water balance studies have assumed all returns of water post irrigation to be to groundwater [18]. Both assumptions represent a gross oversimplification. Instead, the impacts of freshwater withdrawals for irrigation upon surface water and groundwater interactions and surface-subsurface water budgets across the United States are highly localized and depend on factors such as the presence of artificial drainage and efficiency of irrigation method [19, 20]. Determining the proportion of irrigation return flows via surface runoff and subsurface recharge has important implications for C cycling and will require comprehensive country-wide datasets reporting on irrigation efficiency and the impacts of irrigation on the water balance. Whilst this data is currently lacking and thus preventing the robust estimation of net irrigation WD-DIC fluxes, fluxes of DIC returned to groundwater due to leakages from irrigation canals have been estimated. Given that 40% of freshwater withdrawals are done so away from the location of use and that 15% of this water is subsequently leaked from irrigation channels [21], we estimate that irrigation canal leakage will return 0.48 Tg C yr^{-1} to the subsurface, with 37.5% of this flux (0.18 Tg C yr^{-1}) representing a net removal from fresh surface waters (Figure 2).

The use of water for irrigation will also lead to changes in DIC speciation and concentration. For example, freshwater withdrawals can cause increased evaporation [22], which in turn may cause DIC within the remaining water to become more concentrated. However, this may also occur in tandem with the precipitation of carbonate minerals within soils, CO_2 emissions and the utilization of DIC for primary production, that can act to decrease DIC concentrations and the amount of DIC that can be leached to groundwaters or runoff to surface waters [23]. Data relating to these speciation and concentration changes remain spatially limited however, and along with a lack of comprehensive data relating to the impact of freshwater withdrawals upon the US water balance, net irrigation WD-DIC fluxes remain unknown. With irrigation being the largest sector contributing to the gross national-level WD-DIC flux, estimating the net

impact of irrigation withdrawals on DIC cycling has implications for the accurate determination of other sectoral net WD-DIC fluxes and should be a future modelling and research priority.

Public Supply and Domestic

After withdrawal, leakage and outdoor water use have been identified as mechanisms returning fresh water and associated dissolved constituents to the subsurface [24, 25]. Through the use of nationally averaged leakage (16%) [26] and outdoor water use (33%) rates [27], leakage from public supply distribution pipes and outdoor water use at domestic residences resulted in a net public supply WD-DIC_{sw} flux of 0.43 Tg C yr^{-1}. This study has assumed any remaining water would be returned exclusively to surface water environments . Approximately 95% of the remaining public supply and domestic WD-DIC_{gw} and WD-DIC_{sw} fluxes will be returned to wastewater treatment plants and subsequently be released into a surface water environment [28], resulting in a combined net public supply and domestic WD-DIC_{gw} flux of 0.75 Tg C yr^{-1} (Figure 2).

It has been estimated that municipal WWTPs process around $6.06x10^{13}$ L wastewater yr^{-1} [29]. This volume exceeds the volume of water initially withdrawn for public supply and domestic uses and is likely due to processes including inflow of groundwater and precipitation into potable and wastewater distribution networks and inter-basin water transfers [30]. WWTP effluents are returned to surface water environments with elevated DIC concentrations [10, 31] that can contribute to increased DIC exports and acidification downstream [32-35]. Assuming bicarbonate concentrations from US WWTP discharges are analogous to DIC concentrations [36], we estimate a median WWTP effluent DIC concentration of 48 mg C L^{-1}. We therefore estimate a total WWTP effluent DIC flux of 2.9 Tg C yr^{-1}. Fresh water used for public supply is likely to undergo a vast range of treatment processes that may impact DIC speciation and retention, such as pH adjustment [37]. Similarly, in-pipe processes such as the precipitation of carbonates within pipes may retain DIC and prevent it from being further transported downstream. Data relating to these processes is limited across the United States and thus limits the accurate determination of a net WD-DIC_{sw} flux at this time.

Thermoelectric

Similarly to how reservoirs and dams for public supply and irrigation can retain fresh waters [38], the recirculation of water within recirculating thermoelectric cooling plants can retain both fresh water and associated DIC. The consumptive use of water within once-through and recirculating thermoelectric plants were assumed to be 0% and 100%, respectively [39]. The proportion of total freshwater thermoelectric

withdrawals that were used within recirculating plants across the US [1] was determined and applied to gross county-level thermoelectric WD-DIC_{gw} and WD-DIC_{sw} estimates. The recirculation of water within these plants contributed 0.024 Tg C yr^{-1} and 0.17 Tg C yr^{-1} to net thermoelectric WD-DIC_{gw} and WD-DIC_{sw} fluxes, respectively. Return flows of water used within recirculating thermoelectric plants were assumed to be exclusively to surface water environments [30], therefore contributing a further 0.008 Tg C yr^{-1} to the total net WD-DIC_{gw} flux (0.032 Tg C yr^{-1}; Figure 2). The impact that elevated once-through thermoelectric plant effluent temperatures can have on CO_2 release is discussed in Supplementary Note 3.

Industry

Return flows of water used for the industrial sector was assumed to be exclusively to surface water environments [30, 40], therefore resulting in a net industry WD-DIC_{gw} flux that is equivalent to its gross flux (0.21 Tg C yr^{-1}; Figure 2). Water used within industry is subjected to a vast range of process that are likely to change water chemistry (e.g. evaporation, treatment and increased temperatures) and thus impact DIC retention and the net irrigation WD-DIC_{sw} flux. Data relating to these changes is not available on a national level, limiting our ability to determine net industrial WD-DIC fluxes.

Livestock

It was assumed that 100% of water used for livestock were consumed [20, 41], thus resulting in net livestock WD-DIC_{sw} and WD-DIC_{gw} fluxes that are equivalent to gross WD-DIC_{gw} and WD-DIC_{sw} fluxes (0.11 and 0.033 Tg C yr^{-1}, respectively; Figure 2).

Aquaculture

Most withdrawals of fresh water for aquaculture are used within flow-through aquaculture systems that return water exclusively to surface water environments with negligible consumption [42]. In contrast, water used within aquaculture ponds was assumed to be consumptive [43]. The total volume of water stored in these ponds (1.14×10^{12} L yr^{-1}) was estimated from known pond surface area and average depth across the United States (area = 5.72×10^{8} m^2, depth = 2 m) [44, 45]. The proportion of water therefore stored in ponds, in comparison to total freshwater aquaculture withdrawals (9.83×10^{12} m^3) [1], is 12%. Applying this proportion to total gross freshwater aquaculture WD-DIC_{gw} and WD-DIC_{sw} fluxes resulted in net aquaculture WD-DIC_{gw} and WD-DIC_{sw} fluxes of 0.01 and 0.025 Tg C yr^{-1}, respectively. The return of all non-consumed

water to surface water environments increases the net aquaculture WD-DIC_{gw} flux by 0.1 Tg C yr^{-1} (Figure 2).

Mining

Processes such as flowback water and reinjection make determining the fate of freshwater withdrawals used for mining highly complex [46]. Data disclosing the proportion of mining water that is stored or returned to surface water and groundwater environments, as well as any associated changes in DIC concentrations, are currently limited across the United States. Although withdrawals for the mining sector are relatively minor on a large (global and national) spatial scale, they can make major contributions to overall freshwater use on localised scales. Withdrawals are also often within environmentally sensitive locations [47], meaning they may have an important impact on overall freshwater nutrient cycling within an area. The potential local significance of freshwater withdrawals for local water quality therefore supports the need to further resolve net mining WD-DIC fluxes across actively mining parts of the United States.

Table S2. Sources of data and assumptions adopted to estimate net freshwater withdrawal dissolved inorganic carbon fluxes. Note that no assumptions or data were available to estimate any process associated with net mining fluxes.

Assumption/Data	Value (unit)	Reference
**Irrigation**		
Off-farm withdrawals/leakage of conveyance infrastructure	40%/15%	Hrozencik, Potter and Wallander [21]
**Thermoelectric**		
Consumptive use of water in recirculating/once-through plants	100%/0%	Dieter, Linsey, Caldwell, Harris, Ivahnenko, Lovelace, Maupin and Barber [1,] Lee, Han, Elgowainy and Wang [39]
Proportion of return flows to surface water	100%	Templin, Herbert, Stalnaker, Horn and Solley [40]
**Public Supply and Domestic**		
National average leakage rate	16%	USEPA [26]
Outdoor water use	33%	USEPA [27]
Proportion of centralized wastewater treatment plant return flows to surface water	95%	USEPA [28]
**Industry**		
Proportion of return flows to surface water	100%	McCarthy, Brogan, Shortridge, Burgholzer, Kleiner and Scott [30]
**Livestock**		
Consumptive use	100%	Döll, Hoffmann-Dobrev, Portmann, Siebert, Eicker, Rodell, Strassberg and Scanlon [20,] Marston, Ao, Konar, Mekonnen and Hoekstra [41]
**Aquaculture**		
Proportion of return flows to surface water	100%	Dieter, Maupin, Caldwell, Harris, Ivahnenko, Lovelace, Barber and Linsey [42]
Average pond depth	2m	Boyd, Lim, de Queiroz Queiroz, Salie and Lorens de Wet [44]
Total aquaculture pond surface area across the US	5.74×10^8 m^2	USDA [45]

Supplementary Note 3. Estimating CO_2 Degassing Due to Elevated Thermoelectric Effluent Temperatures

This section expands on the data and methodology used to estimate the CO_2 degassing associated with increased thermoelectric plant effluent temperatures. It was hypothesized that the elevated temperatures of once-through cooling plant effluents may result in reduced CO_2 solubility and thus additional CO_2 degassing. To test this hypothesis, the THINCARB model was used to calculate the excess partial pressure of CO_2 ($EpCO_2$) within effluent waters of elevated temperatures. Median county-level thermoelectric plant effluent temperatures, for the year 2015, were calculated using data sourced from the USEPA's online 'Water Pollution Search' tool [36]. These effluent temperatures were then assigned to any of that county's constituent surface water sites that had corresponding alkalinity, pH, altitude, and where possible calcium concentrations, sourced from the USGS Water Quality Portal [48] (see Supplementary Note 1 for more information on the acquisition of these water quality parameters). This assumed that water used within once-through cooling plants was entirely sourced from surface water, based on the fact that over 99.9% of water withdrawals for once-through cooling are from surface water [1].

Model output $EpCO_2$ values were then used to determine excess CO_2 concentrations within effluents ($E[CO_2]_{eff}$) at each site (Section 4.3). County-level CO_2 emissions from thermoelectric effluent degassing were then determined as the product of median county-level $E[CO_2]_{eff}$ and county-level volumes of water returned after use within once-through cooling plants [1]. County-level CO_2 emissions were then summed to give a national-level total of 0.35 Tg CO_2 yr^{-1}.

Supplementary Note 4. Impacts of assuming full atmospheric equilibrium and residual CO_2 supersaturation upon WD-$CO_{2\ gw}$ fluxes

Determining WD-$CO_{2\ gw}$ fluxes relies on the assumptions that groundwater will reach full equilibration with the atmosphere ($pCO_{2\ gw} = pCO_{2\ atm}$) and that the rate of CO_2 degassing from supersaturated groundwater is faster than the rate of groundwaters return to an aquifer after use (equations 5-8 within main text) [9, 49, 50]. To investigate the impact of the above assumptions upon WD-$CO_{2\ gw}$ fluxes, a further analysis was undertaken, whereby groundwater CO_2 was assumed to degas until it reached an equivalent CO_2 supersaturation level of fresh surface water (equations 1-3).

For this validation, county-level emissions of CO_2 due to the degassing of CO_2 supersaturated groundwater withdrawals (WD-$CO_{2\ gw}$, in kg CO_2 yr^{-1}) were estimated as the product of county-level fresh groundwater withdrawal volumes (WD_{gw}, in L yr^{-1}) and median county-level excess CO_2 concentrations of groundwater when in equilibrium with surface water ($E[CO_{2\ gw\text{-}sw}]$; equation 1). County-level excess concentrations of CO_2 in groundwater samples ($E[CO_{2\ gw\text{-}sw}]$), in mg CO_2 L^{-1}, were determined as the difference between $CO_{2\ gw}$ concentrations and median state-level surface water CO_2 concentrations ($CO_{2\ sw}$) [51] (equation 3). $CO_{2\ gw}$ values were estimated using $pCO_{2\ [gw]}$ values (equation 2 and main text equation 7). Median state-level $E[CO_{2\ gw\text{-}sw}]$ concentrations were assigned to any constituent counties without a county-level concentration (n=2,084 counties). The national WD-$CO_{2\ gw}$ flux was determined through the summation of county-level estimates.

$$\text{WD-}CO_{2\ gw} = WD_{gw} \times E[CO_{2\ gw\text{-}sw}] \quad (1)$$

$$CO_{2\ [gw]} = pCO_{2\ [gw]} \times 1000 \times 44.01 \quad (2)$$

$$E[CO_{2\ gw\text{-}sw}] = CO_{2\ gw} - CO_{2\ sw} \quad (3)$$

Assuming equilibrium between groundwater and surface water resulted in a national-level WD-CO_2 flux of 3.3 Tg CO_2 yr^{-1}, which is 8% lower than the initial estimate made using the assumption of full equilibrium with the atmosphere (3.6 Tg CO_2 yr^{-1}). Whilst neither methodology presented in this work are able to utilise data that capture the complexity of localized conditions controlling CO_2 degassing from groundwater (such as turbulent mixing), the similarity of the national-level WD-$CO_{2\ gw}$ flux (i.e. within 10% of each other) suggests that this methodology is robust and provides a good opportunity to begin estimating the sub-national scale patterns in this emissions source.

Supplementary References

(1) Dieter, C. A.; Linsey, K. S.; Caldwell, R. R.; Harris, M. A.; Ivahnenko, T. I.; Lovelace, J. K.; Maupin, M. A.; Barber, N. L. Estimated Use of Water in the United States County-Level Data for 2015. 2018. DOI: 10.5066/F7TB15V5

(2) Stets, E. G.; Striegl, R. G.; Wickland, K. P.; Dornblaser, M.; Foks, S. Dissolved Carbon Export by Large River Systems Is Influenced by Source Area Heterogeneity. *Global Biogeochemical Cycles* **2023**, *37* (2), e2022GB007392. DOI: 10.1029/2022GB007392.

(3) Potter, L.; Xu, Y. J. Variability of Carbon Export in the Lower Mississippi River during an Extreme Cold and Warm Year. *Water* **2022**, *14* (19). DOI: 10.3390/w14193044.

(4) Steyaert, J. C.; Condon, L. E.; W.D. Turner, S.; Voisin, N. ResOpsUS, a dataset of historical reservoir operations in the contiguous United States. *Scientific Data* **2022**, *9* (1), 34. DOI: 10.1038/s41597-022-01134-7.

(5) Song, C.; Gardner, K. H.; Klein, S. J. W.; Souza, S. P.; Mo, W. Cradle-to-grave greenhouse gas emissions from dams in the United States of America. *Renewable and Sustainable Energy Reviews* **2018**, *90*, 945-956. DOI: 10.1016/j.rser.2018.04.014.

(6) Li, C.; Wang, Y.; Yi, Y.; Wang, X.; Augusto Guimarães Santos, C.; Liu, Q. A review of reservoir carbon Cycling: Key Processes, influencing factors and research methods. *Ecological Indicators* **2024**, *166*, 112511. DOI: 10.1016/j.ecolind.2024.112511.

(7) Jarvie, H. P.; King, S. M.; Neal, C. Inorganic carbon dominates total dissolved carbon concentrations and fluxes in British rivers: Application of the THINCARB model – Thermodynamic modelling of inorganic carbon in freshwaters. *Science of The Total Environment* **2017**, *575*, 496-512. DOI: 10.1016/j.scitotenv.2016.08.201.

(8) Rounds, S. A.; Wilde, F. D. Chapter A6. Section 6.6. Alkalinity and acid neutralizing capacity. *Techniques of Water-Resources Investigations* **2012**. DOI: 10.3133/twri09A6.6.

(9) Macpherson, G. L. CO2 distribution in groundwater and the impact of groundwater extraction on the global C cycle. *Chemical Geology* **2009**, *264* (1), 328-336. DOI: 10.1016/j.chemgeo.2009.03.018.

(10) Regnier, P.; Friedlingstein, P.; Ciais, P.; Mackenzie, F. T.; Gruber, N.; Janssens, I. A.; Laruelle, G. G.; Lauerwald, R.; Luyssaert, S.; Andersson, A. J.; Arndt, S.; Arnosti, C.; Borges, A. V.; Dale, A. W.; Gallego-Sala, A.; Goddéris, Y.; Goossens, N.; Hartmann, J.; Heinze, C.; Ilyina, T. Joos, F.; LaRowe, D. E.; Leifeld, J.; Meysman, F. J. R.; Munhoven, G.; Raymond, P. A.; Spahni, R.; Suntharalingam, P.; Thullner, M. Anthropogenic perturbation of the carbon fluxes from land to ocean. *Nature Geoscience* **2013**, *6* (8), 597-607. DOI: 10.1038/ngeo1830.

(11) Vesper, D. J.; Edenborn, H. M.; Billings, A. A.; Moore, J. E. A Field-Based Method for Determination of Dissolved Inorganic Carbon in Water Based on CO(2) and Carbonate Equilibria. *Water Air Soil Pollut* **2015**, *226*. DOI: 10.1007/s11270-015-2348-z From NLM. Eberts, S. M.; Wagner, C. R.; Woodside, M. D. *Water priorities for the nation—The U.S. Geological Survey next generation water observing system*; Reston, VA, 2019. https://pubs.usgs.gov/publication/fs20193046DOI: 10.3133/fs20193046.

(12) Di Lorenzo, P. *usmap: US Maps Including Alaska and Hawaii*. 2022. https://CRAN.R-project.org/package=usmap (accessed 2022).

(13) U.S. Geological Survey. *Principal Aquifers of the 48 Conterminous United States, Hawaii, Puerto Rico, and the U.S. Virgin Islands: U.S. Geological Survey data release*. 2003. DOI:10.5066/P9Y2HOUJ. (accessed 5th January 2024).

(14) U.S. Geological Survey, *Annual NLCD Collection 1 Science Products: U.S. Geological Survey data release*. 2024. DOI:10.5066/P94UXNTS

(15) McCafferty, A. E., San Juan, C.A., Lawley, C.J.M., Graham, G.E., Gadd, M.G., Huston, D.L., Kelley, K.D., Paradis, S., Peter, J.M., and Czarnota, K. *National-scale geophysical, geologic, and mineral resource data and grids for the United States, Canada, and Australia: Data in support of the tri-national Critical Minerals Mapping Initiative: U.S. Geological Survey data release*. 2023. https://doi.org/10.5066/P970GDD5 (accessed 5th January 2025).

(16) Caldwell, P. V.; Sun, G.; McNulty, S. G.; Cohen, E. C.; Moore Myers, J. A. Impacts of impervious cover, water withdrawals, and climate change on river flows in the conterminous US. *Hydrol. Earth Syst. Sci.* **2012**, *16* (8), 2839-2857. DOI: 10.5194/hess-16-2839-2012.
(17) Duan, K.; Sun, G.; Caldwell, P. V.; McNulty, S. G.; Zhang, Y. Implications of Upstream Flow Availability for Watershed Surface Water Supply across the Conterminous United States. *JAWRA Journal of the American Water Resources Association* **2018**, *54* (3), 694-707. DOI: https://doi.org/10.1111/1752-1688.12644 (acccessed 2024/06/06).
(18) de Graaf, I. E. M.; van Beek, L. P. H.; Wada, Y.; Bierkens, M. F. P. Dynamic attribution of global water demand to surface water and groundwater resources: Effects of abstractions and return flows on river discharges. *Advances in Water Resources* **2014**, *64*, 21-33. DOI: 10.1016/j.advwatres.2013.12.002.
(19) Leng, G.; Huang, M.; Tang, Q.; Gao, H.; Leung, L. R. Modeling the Effects of Groundwater-Fed Irrigation on Terrestrial Hydrology over the Conterminous United States. *Journal of Hydrometeorology* **2014**, *15* (3), 957-972. DOI: 10.1175/JHM-D-13-049.1.
(20) Döll, P.; Hoffmann-Dobrev, H.; Portmann, F. T.; Siebert, S.; Eicker, A.; Rodell, M.; Strassberg, G.; Scanlon, B. R. Impact of water withdrawals from groundwater and surface water on continental water storage variations. *Journal of Geodynamics* **2012**, *59-60*, 143-156. DOI: 10.1016/j.jog.2011.05.001.
(21) Hrozencik, R. A.; Potter, N. A.; Wallander, S. *A National Estimate of Irrigation Canal Lining and Piping Water Conservation*; National Bureau of Economic Research, 2022. DOI: 10.3386/w30123.
(22) Haddeland, I.; Skaugen, T.; Lettenmaier, D. P. Anthropogenic impacts on continental surface water fluxes. *Geophysical Research Letters* **2006**, *33* (8). DOI: 10.1029/2006GL026047 (acccessed 2024/06/04).
(23) Ortiz, A. C.; Jin, L.; Ogrinc, N.; Kaye, J.; Krajnc, B.; Ma, L. Dryland irrigation increases accumulation rates of pedogenic carbonate and releases soil abiotic CO2. *Scientific Reports* **2022**, *12* (1), 464. DOI: 10.1038/s41598-021-04226-3.
(24) Flint, E. M.; Ascott, M. J.; Gooddy, D. C.; Stahl, M. O.; Surridge, B. W. J. Water Supply Processes Are Responsible for Significant Nitrogen Fluxes Across the United States. *Global Biogeochemical Cycles* **2022**, *36* (9). DOI: 10.1029/2022GB007340 (acccessed 2022/09/09).
(25) Flint, E. M.; Ascott, M. J.; Gooddy, D. C.; Stahl, M. O.; Surridge, B. W. J. Watermains Leakage and Outdoor Water Use Are Responsible for Significant Phosphorus Fluxes to the Environment Across the United States. *Global Biogeochemical Cycles* **2023**, *37* (3), e2022GB007614. DOI: 10.1029/2022GB007614 (acccessed 2023/08/20).
(26) USEPA. *Water audits and water loss control for public water systems (EPA 816/F/13/002)*; Office of Water 2013. https://www.epa.gov/sites/default/files/2015-04/documents/epa816f13002.pdf.
(27) USEPA. *Outdoor Water Use in the United States*; 29th April; 2017. https://19january2017snapshot.epa.gov/www3/watersense/pubs/outdoor.html.
(28) USEPA. *Clean Watersheds Needs Survey 2012 - Report to Congress*; 2016. https://www.epa.gov/sites/default/files/2015-12/documents/cwns_2012_report_to_congress-508-opt.pdf.
(29) Arabi, S. M. S.; Alicata, J.; Hanigan, D.; Hiibel, S. R. Capturing atmospheric carbon dioxide by depleting inorganic carbon in municipal wastewater. *International Journal of Greenhouse Gas Control* **2021**, *111*, 103472. DOI: 10.1016/j.ijggc.2021.103472.
(30) McCarthy, M.; Brogan, C.; Shortridge, J.; Burgholzer, R.; Kleiner, J.; Scott, D. Estimating Facility-Level Monthly Water Consumption of Commercial, Industrial, Municipal, and Thermoelectric Users in Virginia. *JAWRA Journal of the American Water Resources Association* **2022**, *58* (6), 1358-1376. DOI: 10.1111/1752-1688.13037 (acccessed 2023/07/18).
(31) Ator, S. W.; Miller, O. L.; Saad, D. A. Effects of return flows on stream water quality and availability in the Upper Colorado, Delaware, and Illinois River Basins. *PLOS Water* **2022**, *1* (7), e0000030. DOI: 10.1371/journal.pwat.0000030.
(32) Hossler, K.; Bauer, J. E. Amounts, isotopic character, and ages of organic and inorganic carbon exported from rivers to ocean margins: 2. Assessment of natural and anthropogenic controls. *Global Biogeochemical Cycles* **2013**, *27* (2), 347-362, 10.1002/gbc.20034. DOI: 10.1002/gbc.20034 (acccessed 2023/06/20).

(33) Alshboul, Z.; Encinas-Fernández, J.; Hofmann, H.; Lorke, A. Export of Dissolved Methane and Carbon Dioxide with Effluents from Municipal Wastewater Treatment Plants. *Environmental Science & Technology* **2016**, *50* (11), 5555-5563. DOI: 10.1021/acs.est.5b04923.
(34) Barnes, R. T.; Raymond, P. A. The contribution of agricultural and urban activities to inorganic carbon fluxes within temperate watersheds. *Chemical Geology* **2009**, *266* (3), 318-327. DOI: 10.1016/j.chemgeo.2009.06.018.
(35) Yang, X.; Xue, L.; Li, Y.; Han, P.; Liu, X.; Zhang, L.; Cai, W.-J. Treated Wastewater Changes the Export of Dissolved Inorganic Carbon and Its Isotopic Composition and Leads to Acidification in Coastal Oceans. *Environmental Science & Technology* **2018**, *52* (10), 5590-5599. DOI: 10.1021/acs.est.8b00273.
(36) USEPA. *Enformcement and Compliance History Online (ECHO). Water Pollutant Loading Tool.* Environmental Protection Agency, 2023. https://echo.epa.gov/trends/loading-tool/get-data/custom-search/ (accessed 2023 April).
(37) Arnold, R. B.; Rosenfeldt, B.; Rhoades, J.; Owen, C.; Becker, B. *Corrosion Control: Results from a National Survey*; Hazen and Sawyer, 2019. https://www.hazenandsawyer.com/publications/evolving-utility-practices-and-experiences-with-corrosion-control-results-f/ (accessed 12th July 2022).
(38) Yang, H.; Piao, S.; Huntingford, C.; Ciais, P.; Li, Y.; Wang, T.; Peng, S.; Yang, Y.; Yang, D.; Chang, J. Changing the retention properties of catchments and their influence on runoff under climate change. *Environmental Research Letters* **2018**, *13* (9), 094019. DOI: 10.1088/1748-9326/aadd32.
(39) Lee, U.; Han, J.; Elgowainy, A.; Wang, M. Regional water consumption for hydro and thermal electricity generation in the United States. *Applied Energy* **2018**, *210*, 661-672. DOI: 10.1016/j.apenergy.2017.05.025.
(40) Templin, W.; Herbert, R. A.; Stalnaker, C. B.; Horn, M.; Solley, W. B. *Water Use: National Handbook of Recommended Methods for Water Data Acquisition*; U.S. Geological Survey, 1980. DOI: https://pubs.usgs.gov/chapter11/.
(41) Marston, L.; Ao, Y.; Konar, M.; Mekonnen, M. M.; Hoekstra, A. Y. High-Resolution Water Footprints of Production of the United States. *Water Resources Research* **2018**, *54* (3), 2288-2316. DOI: 10.1002/2017WR021923 (acccessed 2023/08/07).
(42) Dieter, C. A.; Maupin, M. A.; Caldwell, R. R.; Harris, M. A.; Ivahnenko, T. I.; Lovelace, J. K.; Barber, N. L.; Linsey, K. S. *Estimated use of water in the United States in 2015*; Reston, VA, 2018. DOI: 10.3133/cir1441.
(43) Gephart, J. A.; Troell, M.; Henriksson, P. J. G.; Beveridge, M. C. M.; Verdegem, M.; Metian, M.; Mateos, L. D.; Deutsch, L. The `seafood gap' in the food-water nexus literature—issues surrounding freshwater use in seafood production chains. *Advances in Water Resources* **2017**, *110*, 505-514. DOI: 10.1016/j.advwatres.2017.03.025.
(44) Boyd, C. E. B.; Lim, C. L.; de Queiroz Queiroz, J. F.; Salie, K. S.; Lorens de Wet, W. *Best management practices for responsible aquaculture*; 2008. https://www.dffe.gov.za/sites/default/files/legislations/guidebestmanagementpractice_responsibleaquaculture.pdf.
(45) USDA. *2018 Census of Aquaculture*; AC-17-SS-2; 2019. https://www.nass.usda.gov/Publications/AgCensus/2017/Online_Resources/Aquaculture/Aqua.pdf.
(46) Kondash, A. J.; Albright, E.; Vengosh, A. Quantity of flowback and produced waters from unconventional oil and gas exploration. *Sci Total Environ* **2017**, *574*, 314-321. DOI: 10.1016/j.scitotenv.2016.09.069 From NLM. Veil, J. *US Produced Water Volumes and Management Practices in 2017*; 2020. https://www.gwpc.org/wp-content/uploads/2020/02/pw_report_2017___final.pdf.
(47) Miranda, M.; Sauer, A. *Mine the Gap: Connecting Water Risks and Disclosure in the Mining Sector*; Washington, DC., 2010. http://pdf.wri.org/working_papers/mine_the_gap.pdf.
(48) WQP. *Water Quality Portal*. 2023. https://www.waterqualitydata.us/ (accessed 2023 5th April).
(49) Deirmendjian, L.; Loustau, D.; Augusto, L.; Lafont, S.; Chipeaux, C.; Poirier, D.; Abril, G. Hydro-ecological controls on dissolved carbon dynamics in groundwater and export to streams in a temperate pine forest. *Biogeosciences* **2018**, *15* (2), 669-691. DOI: 10.5194/bg-15-669-2018.

(50) Cole, J. J.; Prairie, Y. T.; Caraco, N. F.; McDowell, W. H.; Tranvik, L. J.; Striegl, R. G.; Duarte, C. M.; Kortelainen, P.; Downing, J. A.; Middelburg, J. J.; Melack, J. Plumbing the Global Carbon Cycle: Integrating Inland Waters into the Terrestrial Carbon Budget. *Ecosystems* **2007**, *10* (1), 172-185. DOI: 10.1007/s10021-006-9013-8.
(51) Toavs, T. R.; Hasler, C. T.; Suski, C. D.; Midway, S. R. A 30-year dataset of CO2 in flowing freshwaters in the United States. *Scientific Data* **2023**, *10* (1), 20. DOI: 10.1038/s41597-022-01915-0.
